# Fermented Rice Bran: A Promising Therapeutic Agent Against High‐Fat Diet‐Induced Metabolic Disorders

**DOI:** 10.1002/fsn3.71439

**Published:** 2026-01-09

**Authors:** Afroza Sultana, Md. Ruhul Amin, Md. Omar Faruque, Muhammad Ali Siddiquee, Md. Zakir Hossain Howlader, Md. Alauddin

**Affiliations:** ^1^ Department of Nutrition and Food Technology Jashore University of Science and Technology Jashore Bangladesh; ^2^ The Aristocrat Food Ltd. Dhaka Bangladesh; ^3^ Department of Biochemistry and Molecular Biology University of Dhaka Dhaka Bangladesh; ^4^ Department of Biochemistry and Molecular Biology Jashore University of Science and Technology Jashore Bangladesh

**Keywords:** cognitive function, diabetes, dyslipidemia, hepatic gene expression, nutritional traits of fermented rice bran (FRB)

## Abstract

The increasing consumption of a high‐fat diet (HFD) triggers metabolic diseases such as obesity, diabetes, dyslipidemia, and MAFLD (metabolic dysfunction‐associated fatty liver diseases). This situation demands natural and healthy therapeutic diets. Rice bran is a widely available, nutrient‐dense by‐product of rice. This study aimed to explore the compositional profile of optimized solid‐phase fermented rice bran (FRB) and nonfermented rice bran (NFRB) and, subsequently, its effect on HFD‐induced obese rats. We found that the fermentation process improved the nutritional quality and antioxidant properties of rice bran, and FRB supplementation (8 weeks) decreased body weight gain by 3.5%, blood glucose levels by 2.03 mmol/L, and liver fat. However, FRB administration improved cognitive function, while the improvement in lipid profile was associated with the downregulation of hepatic genes (G6PC, FASN, and HMGCR) expression. Additionally, liver tissue staining suggests that FRB supplementation can improve liver morphology by reducing sinusoidal dilation, fat accumulation, and necrosis. Our results demonstrate that FRB could be effectively used to treat and prevent lifestyle‐related diseases.

## Graphical abstract

Fermented rice bran (FRB) has nutritional quality and antioxidant properties higher than nonfermented rice bran (NFRB). Supplementation of FRB for 8 weeks decreased body weight gain by 3.5%, blood sugar levels by 2.03 mmol/L, and liver fat, as well as improved cognitive function and lipid profile, which was associated with downregulation of hepatic genes (G6PC, FASN, and HMGCR) expression. FRB could be a source of functional foods and effectively used to treat and prevent lifestyle‐related diseases.

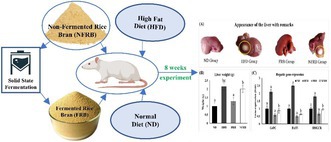

**Abbreviations**
FRB: fermented rice bran
HFD: high‐fat diet
ND: normal diet
NFRB: nonfermented rice bran

## 1 Introduction

Globally, energy‐dense high‐fat diet (HFD) intake is increasing rapidly (WHO 2021). The consumption of a high‐fat diet is strongly linked to lifestyle‐related diseases, including obesity, type 2 diabetes, metabolic dysfunction‐associated fatty liver disease (MAFLD), hypertension, hyperlipidemia, and cognitive impairments. These noncommunicable diseases now account for over half of the global disease burden and contribute to 70% of global deaths (Naghavi et al. 2017; WHO 2016). In the modern era of medical science, lifestyle‐related diseases can be managed through the administration of various pharmaceutical interventions. It has been reported that prolonged use of synthesized drugs as a treatment for noncommunicable diseases (NCDs) may result in adverse effects for patients (Mirjalili et al. 2022; WHO 2023). Numerous studies suggest that lifestyle‐related diseases can be prevented through modifications to dietary and physical activity habits. Increasingly, consumers are seeking specific functional foods over synthetic drugs to avoid undesirable side effects often associated with costly chemical drugs. In recent years, there has been significant exploration and investigation into the biological activities, nutritional values, and potential health benefits of numerous medicinal and edible plants. Their effectiveness in preventing and alleviating lifestyle‐related disorders has been extensively validated (Acquaviva et al. 2021; Edward Mashau and Eugenia Ramashia 2021).

Rice, a staple food for over 50% of the global populace, is cultivated by over 100 countries, with Asia accounting for 90% of worldwide production. Rice bran, a by‐product of rice milling, is estimated to have an annual global production of 76 million tons (T. Kahlon 2009). At present, rice bran is mainly used as animal and poultry feed, with a tiny proportion also being extracted for bran oil and used as a dietary supplement or component of the microbiological medium. Additionally, rice bran contains 11.0%–17.0% protein, 12.0%–22.0% fat, 6.0%–14.0% fiber, 8.0%–17.0% ash, and 10.0%–15.0% moisture, as well as anthocyanins in red and black rice bran (Chen et al. 2012; Pengkumsri et al. 2015). Moreover, rice bran extracts contain several bioactive compounds that reportedly possess anti‐inflammatory (Limtrakul et al. 2016), antioxidant (Surarit et al. 2015), and anti‐diabetic properties (Boue et al. 2016; Tantipaiboonwong et al. 2017). Furthermore, rice bran is a great source of antioxidants like gamma‐oryzanol, polyphenols, tocopherols, and tocotrienols, which can stop cells from oxidizing and damaging DNA and tissue. These compounds are increasingly recommended to be included in diets due to their beneficial effects on human metabolism, which include lowering cholesterol, improving cardiovascular health, and having anticancer activity. Several studies have been conducted to assess the potential of rice bran for human consumption due to its poor commercial value (Christ‐Ribeiro et al. 2019; Massarolo et al. 2017; Peanparkdee and Iwamoto 2019). However, even though rice bran is widely available, there are several criticisms about the palatability and digestibility of this material for food formulation, and little research has focused on creating products from rice bran. In vitro enzyme‐digested rice bran is effective against lifestyle‐related diseases in rat and mouse models (Candiracci et al. 2014; Justo et al. 2013). The rice bran protein hydrolysates improved diabetic mellitus in an insulin‐resistant mouse model (Boonloh et al. 2015). The beneficial evidence of the chronic disease‐fighting properties of rice bran has advanced the development of rice bran products for human health use as a functional food and a dietary management pattern (Ryan et al. 2011).

Varieties of fermentation techniques, such as liquid fermentation and solid‐state fermentation, have been used in biotechnological applications to enhance the nutritional and functional values of rice bran to make it more beneficial in the management of lifestyle‐related diseases. Liquid fermentation or submerged fermentation (SmF) involves the growth of microorganisms in a liquid medium rich in nutrients and with a high oxygen concentration (aerobic conditions) (Belorkar and Jogaiah 2022). However, this technique has an economic disadvantage. Aeration and agitation are expensive, and the media used in preparing the fermentation are often costly. On the other hand, solid‐state fermentation (SSF) occurs in the absence of free water, and the substrate contains enough moisture to ensure microbial growth (Belorkar and Jogaiah 2022). Solid‐state fermentation (SSF) has gained significant attention in recent years due to its potential in developing sustainable industrial bioprocesses. It requires less energy while still achieving high production yields, produces minimal wastewater that reduces the risk of microbial contamination, eliminates common issues such as foaming, supports eco‐friendly practices by using low‐cost agro‐industrial by‐products as carbon sources, and keeps operational costs low (Cano y Postigo et al. 2021). Ultimately, SSF becomes a cost‐effective and environmentally sustainable alternative to conventional fermentation methods. Cereal brans are good sources of substrates. The fermentation process enhances the bioavailability of vitamins and reduces anti‐nutritional factors like phytates, improving nutrient absorption. Fermented rice bran (FRB) significantly increases the levels of beneficial bacteria, such as Lactobacillus and Bifidobacterium, which are crucial for gut health (Demissie et al. 2020). Previous studies have shown that dual fermentation of rice bran with *S. cerevisiae* has anti‐stress effects (Kim et al. 2002). Furthermore, polysaccharides extracted from rice bran have shown anticancer and anti‐defective effects by regulating immune responses, whereas a water extract from fermented rice bran has shown an anti‐photoaging effect (Choi et al. 2014; Kim et al. 2007). Moreover, different organic compounds isolated from rice bran have been shown to have hypoglycemic effects in a mouse model of type 2 diabetes condition (Jung et al. 2007). Additionally, brown rice fermented with *A. oryzae* can suppress sodium dextran sulfate‐induced colitis (Kataoka et al. 2008). It has been shown in other studies that rice bran protein fractions improve glucose and lipid metabolism in animal models of metabolic syndrome (Ardiansyah et al. 2007; Strahorn et al. 2005). Thus, the nutrigenetic effects of fermented rice bran (FRB) on high‐fat diet‐induced diabetic conditions have been examined in only a few studies. As many authors have demonstrated, solid‐state fermentation is an alternative method for improving rice bran's protein, phytochemical, and fiber content as well as its palatability and digestibility while minimizing problems (Christ‐Ribeiro et al. 2019; Utama‐ang et al. 2017).

However, to date, fermented rice bran's whole nutritional traits and effects on high‐fat diet‐induced obese rats have not been fully elucidated. To the best of our knowledge, this is the first study of fermented rice bran's whole nutritional profile exploration and administration to an in vivo model to explore general physiological conditions, the molecular mechanisms of the reduction of obesity, diabetes, and fatty liver, which is also examined with the regulation of hepatic genes (G6PC, FASN, and HMGCR) expression.

## 2 Materials and Methods

### 2.1 Preparation of the Sample and Fermentation Technique Optimization

Rice bran was collected from the Bangladesh Rice Research Institute immediately after milling. Then it was stabilized at 121°C for 15 min within 24 h. Stabilized rice bran was defatted by using organic solvent (sample: n‐hexane; 1:3) extraction through a rotary evaporator (model no. NGG3000). A stock of a probiotic blend of *Lactobacillus acidophilus* , *Lactobacillus bulgaricus* , and *Bifidobacterium bifidum* solution and nutrient broth were prepared. Firstly, 0.5 g of pure probiotic was added to 10 mL of cooled, sterile water. 0.5 g of pure probiotic contains *Lactobacillus acidophilus* (2 billion), *Lactobacillus bulgaricus* (1 billion), and *Bifidobacterium bifidum* (1 billion); thus, the ratio is 2:1:1. Then it was transferred to the nutrient broth and incubated at 37°C at 180 rpm for 48 h in a test tube through a shaking incubator (Biobase, BJPS‐1038). This inoculated substrate was used for fermentation at 37°C for 72 h. All steps were performed under aseptic conditions to prevent contamination. All glassware, nutrient broth, and other accessories were sterilized prior to use. Inoculum preparation and mixing were carried out in a laminar flow cabinet, and fermentation was conducted in sterilized containers. Additionally, we optimized various combinations of samples and probiotics, employing different treatments during fermentation. Technique 1 involved mixing raw rice bran with a probiotic blend, followed by incubation at 37°C for 7 days. In Technique 2, the same mixture was autoclaved before incubation. Technique 3 utilized only raw rice bran, incubated at 37°C for 7 days. In Technique 4, raw rice bran was mixed with previously grown bacteria and then incubated. Technique 5 involved autoclaving the mixture of raw rice bran and previously grown bacteria before incubation at 37°C for 7 days. Then the freshly prepared fermented rice bran (FRB) was dried, finely blended, and preserved at 4°C in a sterile container for further analysis. All of the reagents used for this study were analytical grade and purchased from Sigma‐Aldrich and Loba Chemie, India.

### 2.2 Nutritive Traits and Antioxidant Capacities

#### 2.2.1 Proximate Composition

Nonfermented rice bran (NFRB) samples were used as a control. After fermentation, the nutritional components of all rice bran samples (fermented and nonfermented control) were analyzed according to the following guide. Proximate analysis was carried out according to the procedure of the Association of Official Analytical Chemists (AOAC 1990) for moisture, ash, crude fiber, and crude protein content. The carbohydrate was calculated by the difference method (AOAC 1990) by subtracting the sum (g/100 g) of crude protein, crude fat, ash, and fiber from 100 g. The caloric value was determined based on the Atwater factor (Maclean et al. 2003). All of the measures taken were triplicated for assurance of reproducibility and accuracy.

#### 2.2.2 Amino Acid Profile

To analyze amino acids, a 1‐g sample of finely ground powder was hydrolyzed in a sealed test tube with 6 M HCl at 110°C for 48 h under a nitrogen atmosphere to prevent oxidative degradation of amino acids. The resulting solution was used for amino acid analysis using the amino acid analyzer (High‐Speed Amino Acid Analyzer LA8080 AminoSAAYA, Japan). This method is a slightly modified method described in another study (Dai et al. 2014). All analyses were performed in triplicate, and data were statistically analyzed using Student's *t*‐test at a significance level of *p* < 0.05.

#### 2.2.3 Total Phytochemical Evaluation

Total phenolic and tannin content was determined by using the Folin‐Ciocâlteu assay (Makkar 2003). Complete phenolic content was defined as mg of gallic acid equivalent per gram (g), and tannin content was determined as mg of tannic acid equivalent per gram from the respective standard calibration curve. Total flavonoid content was determined as mg of quercetin equivalent (mg/g of dry sample) (Zhishen et al. 1999).

#### 2.2.4 Antioxidant Activity

The determination of total antioxidant activity was estimated by the phospho‐molybdenum assay method. The antioxidant activity was expressed as the number of grams equivalent to ascorbic acid. The DPPH radical‐scavenging activity (%) was plotted against the extract concentration to determine the concentration of extract necessary to decrease DPPH radical‐scavenging by 50%, called IC_50_. Ascorbic acid was used as a positive control standard procedure, as described in the referenced paper (Prieto et al. 1999). All analyses were conducted in triplicate for assurance of data reliability and reproducibility.

#### 2.2.5 Fatty Acid Profile

Gas chromatography, followed by a flame ionization detector (FID), was used for fatty acid analysis. The preparation of fatty acid methyl ester (FAME) and the esterification of fatty acids were prepared by the method described in the referenced paper (Nemzer and Al‐Taher 2023). Petroleum ether extract (50 mg) and 2 M methanolic NaOH solution (4 mL) were combined in a round‐bottomed flask. The solution was then heated to the boiling point with stirring for 15 min. Afterwards, 5 mL of methanolic BF_3_ (14% w/w) was added to the flask, and heating continued for 5 min. Then isooctane (2 mL) was added, stirred for 5 min, and supplemented with a 2 mL saturated sodium chloride solution. Finally, the upper isooctane layer was passed through an anhydrous sodium sulfate column to remove water. The filtrate was collected in a vial, and 1 μL was injected into gas chromatography for FAME analysis. The fatty acid compositions were examined using a gas chromatograph (Trace 1300, Thermo Scientific, PA, USA), a flame ionization detector, and a fused silica capillary column (TR‐FAME, 30 m 0.25 mm 0.25 m film thickness, Thermo Scientific, PA, USA) in a split injection (20:1) technique. Nitrogen was used as the carrier gas at a constant flow rate of 1 mL/min. The injector temperature was 250°C, whereas the initial oven temperature of 150°C was kept for 5 min. After holding the temperature at 200°C for 5 min at a rate of 5°C/min, it was raised to 240°C for 5 min at a rate of 10°C/min. The results of the automated GC software (Chromeleon, version 7.00) were then displayed as relative percentages after the fatty acids were identified using the corresponding fatty acid methyl ester standards (Supelco 37 Component FAME Mix, USA).

#### 2.2.6 Organic Contents Analysis by GC–MS

Samples were extracted using ethanol and water in the same protocol, with constant stirring for 45 min. The sample was then placed in a shaking incubator at 37°C, 225 rpm for 72 h. After filtration and collection of the extract, drying was done at 40°C. The cooled sample was mixed with 5 mL of ethanol, filtered (0.2 μm), and analyzed using a Clarus690 gas chromatograph (PerkinElmer) equipped with a Clarus SQ 8 C mass spectrophotometer. Gas chromatography–mass spectrometry analysis involved a 1 μL sample injected in spitless mode, using pure helium (99.999%) as a carrier gas at a constant flow rate (1 mL/min) for a 40 min run time. EI mode at 70 eV was employed, with an inlet temperature of 280°C and column oven temperature starting at 60°C (for 0 min), raised at 5°C per minute to 240°C, and held for 4 mins. Compound identification was done by comparing them to the NIST database (Zilani et al. 2021). All other chemicals were used as MS grade.

### 2.3 Animal Experiment

Adult male Swiss albino rats were obtained from the International Centre for Diarrheal Disease Research (ICDDR, B), Dhaka, Bangladesh. Initially, rats' body weight was matched, then experimenters were blinded to group assignments, and randomization was carried out by assigning each animal an identification number, after which animals were allocated into the four groups (ND, normal diet; HFD, high‐fat diet; FRB, fermented rice bran; NFRB, nonfermented rice bran; *n* = 6 per group) using a simple random draw method, ensuring that each animal had an equal chance of being allocated to any group. The animals were maintained at 24°C ± 4°C with a 12 h light/dark cycle. They were provided with a standard diet and water ad libitum. The animal experiment protocol received approval from the Institutional Animal Ethical Committee, JUST (Reference No. ERC/FBST/JUST/2022–119).

After 1 week of acclimatization, the initial body weight and glucose level were measured for all rats. The animals were then assigned to an 8 week chronic dietary intervention. The normal diet (ND) provided 10% of total energy from fat, 20% from protein, and 70% from carbohydrates. The high‐fat diet (HFD) provided 50% of total energy from fat, 20% from protein, and 30% from carbohydrates. The FRB and NFRB diets were formulated by supplementing the HFD with 20% (w/w) fermented rice bran and 20% (w/w) nonfermented rice bran, respectively. Diet composition was partially modified from AIN93A. Throughout the study, we recorded the daily consumption of food and water, as well as monitored fecal excretion. Body weight and blood glucose level were measured by using a digital balance (Ansoff Model‐1000) and glucometer (Accu‐Chek Proforma; Roche).

The study assessed cognitive effects by evaluating the time taken to select a diet specific to each rat in a group. Blood samples were obtained via tail vein puncture after a 16 h fast. An oral glucose tolerance test (OGTT) was conducted following an overnight fast (16–18 h). Baseline blood glucose levels were measured in ND, HFD, FRB, and NFRB groups. Subsequently, blood was drawn at 60, 120, and 240 min post‐administration of the oral glucose solution (2 g/kg body weight).

At the end of the experimental period, animals were sacrificed, and serum was obtained through centrifugation at 4000 rpm for 30 min and stored at −20°C for biochemical analysis. Total glucose was assessed using the enzymatic method (glucose‐oxidase peroxidase), and the lipid profile was determined by the colorimetric method with an Ultra ELISA kit. Additional biological samples were stored at −20°C for subsequent analysis.

### 2.4 Determination of Gene Expression

Hepatic tissue from each experimental animal model was collected, and 30–35 mg of the sample underwent slicing for isolation and purification. The homogenized sample was centrifuged using the Monarch Total RNA Miniprep Kit (New England Biolabs Inc., USA) with slight modifications. RT‐PCR was performed with cyanine dye (SYBR) Green, PCR Master Mix (LUNA, New England Biolabs Inc., USA). The relative quantitative gene expression levels of 3‐hydroxy‐3‐methylglutaryl‐CoA (HMG‐CoA) reductase, fatty acid synthase, and glucose‐6‐phosphatase catalytic subunit‐1 in hepatic tissue were determined. Real‐time cycling parameters included initial denaturation (9°C for 5 min), 40 cycles of denaturation at 95°C for 20 s, annealing at 58°C for 20 s, and extension at 68°C for 20 s, followed by a melting curve analysis for the confirmation of the specificity of PCR, followed by Quant Studio‐5 software. Primers' respective sequences were mentioned in the Table S3.

### 2.5 Liver Histology

Briefly, liver tissue samples were sectioned at 5 μm thickness using a Microtome and subsequently stained with Hematoxylin and Eosin (H&E) staining to evaluate hepatic tissue structural changes and fat accumulation. The stained sections were then examined under the confocal microscope. The tissue section was selected using the Zoom navigation tool, and the selected area was snapped and saved as a JPG file. Consequently, the JPG file was opened in the ImageJ (Fiji) program (version 1.54 g, NIH, New York, NY, USA), and 8‐bit binary conversion was done. Then the “Color threshold” tool was applied to remove inter‐hepatocyte structures that did not indicate lipid droplet features of the tissue section area. The selected area was analyzed by using the “Measure” tool. The color threshold was adjusted to the maximum to remove the background signal.

### 2.6 Statistical Analysis

Sample size (*n* = 6) was determined based on various factors, including effect size, Type 1 error, power, direction of effect, statistical tests, and death of animals. Normality of the data set was tested using the Shapiro–Wilk test. Assumptions of the ANOVA test, including data normality, equal variances, and independent samples, were taken into account. The data were subjected to statistical analysis employing a *T* test and one‐way analysis of variance (ANOVA), followed by a post hoc (Tukey's HSD) test. GraphPad Prism Statistical software (version 10) was utilized for these analyses. Statistical significance was determined at a probability value of < 0.05. Statistical significance was expressed using thresholds (*p* < 0.05) rather than reporting exact *p*‐values.

## 3 Results and Discussion

### 3.1 Evaluation of Fermentation Technique

Rice bran contains valuable nutrients, but it spoils quickly due to lipase enzyme activity. Implementing the appropriate fermentation technique can enhance the nutritional attributes of the product through the microbial metabolism pathway. Different ways of solid‐phase fermentation of rice bran were optimized by employing the edible gram‐positive bacteria blend (*Lactobacillus acidophilus, Lactobacillus bulgaricus, Bifidobacterium bifidum*) as a process strategy to improve the nutritional quality of rice bran. Table S1 highlights the comparison among the fermentation techniques by assessing the intrinsic parameters (phenolic content, fiber, fat, protein, and moisture) of fermented rice bran (FRB). Comparing values in Table S1 across techniques, Tech 1 demonstrated the highest phenolic content (206 mg GAE/g dry extract), along with the highest protein (12.6%) and fiber (22%) content, highlighting the efficacy of probiotic solid‐phase fermentation on rice bran nutrition. Tech 1 and 2 fermentations go through freshly prepared microbes, while Tech 4 and 5 fermentations are carried out with previously stored culture at −20°C. Furthermore, both Tech 2 and Tech 5 applied samples are autoclaved before fermentation, which makes a wide range of differences in the reduction of phenolic content, and protein, as well as other variables also showed deteriorating effects. Table S1 results indicate that the Tech 1 fermentation process is the best among all four techniques due to carrying out fermentation without autoclaving with live probiotic blends, and based on nutritional value. We finalized the Tech 1 process for the production of FRB. We compared the NFRB with the FRB produced by the Tech 1 fermentation process in the remaining study.

The solid‐phase fermentation technique simulates the natural growth conditions for microbes by providing moisture to solid substrates without liberated water, which promotes better diffusion and high enzymatic productivity, but can be limited by difficulty in heat removal, mixing, and product recovery at industrial scales compared to submerged fermentations (SmF) (Mattedi et al. 2023; Pandey 2003). Previous studies have shown that solid‐phase fermentation can be effectively utilized in agro‐industrial food by‐products like rice bran to enhance protein, fiber, and phytochemical content, which is mainly due to microbial enzyme secretions during fermentation (Spaggiari et al. 2021). While all aforementioned techniques can bio‐convert substrates, the optimal configurations vary based on specific goals. Microbial growth, enzyme activities, metabolite diffusion, and interatomic networks differ substantially among these fermentation platforms. The Tech 1 fermentation process is a promising bioprocessing method for the increased nutritional and medicinal value of rice bran.

### 3.2 Evaluation of Nutritive Traits and Antioxidant Properties

#### 3.2.1 Proximate Composition

An overall picture of a food's nutritional value may be obtained by analyzing its major components, such as protein, carbohydrate, lipid, ash, moisture, fiber, and energy level, and referring to the result as the nutrient's composition.

Table 1 illustrates the proximate composition and amino acid profile of rice bran, comparing two phases of the sample: before and after the fermentation process.

**TABLE 1:** Assessment of proximate composition and amino acid profiling of rice bran before and after fermentation.

Parameters	Rice bran types	
	NFRB	FRB
Protein (%)	12.77 ± 2.02	16.54 ± 0.61*
Fat (%)	7.27 ± 0.53	8.20 ± 1.08
Fiber (%)	15.70 ± 0.76	22.70 ± 1.12*
Ash (%)	6.50 ± 1.35	10.59 ± 1.44*
Moisture (%)	4.90 ± 0.56	2.06 ± 0.54*
Carbohydrate (%)	53.14 ± 3.51	40.05 ± 1.38
Energy (Kcal/100 g)	391.07 ± 8.48	390.96 ± 6.65
**Amino acids (nmol)**		
Alanine (Ala)	4.07 ± 0.01	2.60 ± 0.05
Arginine (Arg)	0.65 ± 0.02	8.90 ± 3.44
Asparagine (Asn)	0.21 ± 0.01	2.86 ± 0.1*
Aspartate (Asp)	3.88 ± 0.01	0.03 ± 0.05*
Cystine (Cys)	2.40 ± 0.01	29.64 ± 0.31
Glutamate (Glu)	8.08 ± 0.17	8.50 ± 1.05
Glycine (Gly)	4.42 ± 0.02	13.00 ± 0.10*
Histidine (His)	4.27 ± 0.17	6.83 ± 0.05
Isoleucine (Ile)	1.32 ± 0.11	13.40 ± 1.65*
Leucine (Leu)	3.21 ± 0.13	7.02 ± 0.11
Lysine (Lys)	2.49 ± 0.39	0.72 ± 0.55
Methionine (Met)	0.03 ± 0.03	23.44 ± 1.67
Phenylalanine (Phe)	1.37 ± 0.26	14.66 ± 0.50
Proline (Pro)	6.37 ± 4.61	6.86 ± 0.16
Serine (Ser)	1.90 ± 0.66	13.79 ± 0.19*
Threonine (Thr)	2.19 ± 0.43	0.12 ± 0.11
Tryptophan (Trp)	0.23 ± 0.01	0.26 ± 0.03
Tyrosine (Tyr)	1.69 ± 1.50	12.65 ± 1.43*
Valine (Val)	3.75 ± 4.53	18.37 ± 0.33

*Note:* Values expressed as mean ± SD.

Abbreviations: FRB, fermented rice bran; NFRB, nonfermented rice bran.

* *p* < 0.05.

Fermentation of rice bran by following the Tech‐1 protocol led to significant increases in total proteins from 12.77% to 16.54%, lipids from 7.27% to 8.20%, ash content from 6.50% to 10.59%, and fiber from 15.70% to 22.70%. Furthermore, Table 1 illustrates a decrease in carbohydrate content by 13.09% in FRB (fermented rice bran) compared to NFRB (nonfermented rice bran). The improvement seen in protein, ash, and fiber after fermentation of rice bran can be attributed to the biochemical activity and enzyme secretions of the probiotic microbes used in this study. Specifically, the bacteria *L. acidophilus* and *B. bifidum* have proteolytic systems that enhance true protein levels in rice bran (Seyoum et al. 2022). Fiber increases are likely due to the carbohydrases from *L. acidophilus* and *B. bifidum* , which can degrade complex polysaccharides into dietary fiber and oligosaccharides (Crittenden et al. 2002). The dietary fiber content in FRB may be beneficial for increasing fecal bulk and laxation. The microbial degradation of whole‐grain complex carbohydrates can also increase the production of short‐chain fatty acids, which have potential health benefits (Bach Knudsen 2015). Reduced moisture and fat levels indicate enhanced utilization of the substrate by the probiotic blend, aiding their growth and metabolism. Moreover, lower moisture content improves the storage stability of fermented rice bran. This is because the fermented rice bran samples were defatted, resulting in lower fat content compared to the raw rice bran sample. Elevated ash levels indicate improved mineral availability resulting from microbial fermentation. Overall, fermentation is an effective bioprocessing method to enhance the value of rice bran as a functional food or nutraceutical ingredient.

#### 3.2.2 Amino Acid Profile

Amino acid profiling of FRB (fermented rice bran) and NFRB (nonfermented rice bran) is the first report in Bangladesh. Table 1 demonstrates the assessment of amino acids in FRB and NFRB, and (Figure S1A,B) represents the chromatographic view of amino acid analysis in rice bran. The results showed that Fermented rice bran (FRB) was found to be highly enriched in both essential and nonessential amino acids compared to NFRB. Following fermentation, levels of seven essential amino acids (EAA) (histidine, isoleucine, leucine, methionine, phenylalanine, tryptophan, and valine) and 8 nonessential amino acids (NEAA) (cysteine, asparagine, glutamine, glycine, proline, serine, tyrosine, and arginine) showed significant increases (*p* < 0.05) among the 20 proteinogenic amino acids analyzed. Notably, branched‐chain amino acids (leucine, isoleucine, valine) and aromatic amino acids (phenylalanine, tryptophan, tyrosine), which are crucial precursors for neurotransmitter synthesis (Pogson et al. 1989), exhibited elevated levels. After fermentation, the amounts of EAA and NEAA increased to 66.4 nmol and 36.6 nmol. Generally, the fermentation technique significantly improved some critical amino acids through hydrolysis, following mostly deamination and decarboxylation, which are responsible for several significant biological activities. Prior studies indicate amino acids' crucial roles in immune function, digestion, sleep regulation, and maintaining nitrogen balance. For instance, phenylalanine and tryptophan influence appetite, sleep, and mood, while valine and leucine aid muscle repair (Comai et al. 2020; Li et al. 2007; Wu 2009). Additionally, the fermentation process increases the tyrosine amino acid approximately 8 times in FRB than in NFRB. Previous studies state that tyrosine is the precursor of dopamine synthesis. Firstly, tyrosine is converted into L‐DOPA through the enzyme tyrosine hydroxylase, and then L‐DOPA is converted into dopamine (Kühn et al. 2019). Dopamine is the neurotransmitter that enhances cognitive function. Overall, FRB supplementation provides such important amino acids that may be responsible for improving cognitive function, which needs further specific behavioral assessment through an animal model to confirm this statement.

#### 3.2.3 Evaluation of Total Phytochemical Content

Table 2 represents the analytical data for total phenolic content (TPC), total tannin content (TTC), and total flavonoid content (TFC) of NFRB and FRB. Data clearly show that TPC is significantly (*p* < 0.05) higher in FRB (fermented rice bran) than in NFRB (nonfermented rice bran). Phenolic compounds are ubiquitous secondary metabolites in plants. They are known to have antioxidant activity, and the activity of these extracts is likely due to these compounds (Okuda et al. 1994; Tepe et al. 2006). Additionally, the total flavonoid content is approximately double in FRB compared to NFRB. Flavonoids are one class of secondary plant metabolites that are also known as Vitamin P. In addition, flavonoids are readily ingested by humans, and they seem to display important anti‐inflammatory, anti‐allergic, and anti‐cancer activities (Crozier et al. 2006). Those phenolic compounds are nonharmful to human health, and there is an increase in the use of plant foods with high phenolic amounts in the food industry, aiming to improve the quality of foods (Kähkönen et al. 1999). However, TTC is quite similar in both NFRB and FRB.

**TABLE 2:** Phenolic content and antioxidant properties of fermented and nonfermented rice bran.

Phenolic contents	Rice bran types	
	NFRB	FRB
Total phenolic content (mg GAE/g of dry extract)	90.00 ± 0.24	206.40 ± 0.50**
Total tannin content (mg TAE/g of dry extract)	35.00 ± 0.23	39.17 ± 1.08
Total flavonoid content (mg QE/g of dry extract)	1.33 ± 0.29	2.89 ± 0.36*
**Antioxidant activity**		
Total antioxidant activity (mg AAE/g of dry extract)	74.86 ± 0.05	91.00 ± 0.12**
IC50 (mg/mL)	3.17 ± 0.02	2.38 ± 1.14*

*Note:* Values expressed as mean ± SD.

Abbreviations: AAE, ascorbic acid equilibrium; FRB, fermented rice bran; GAE, gallic acid equilibrium; IC, inhibitory concentration; NFRB, nonfermented rice bran; QE, quercetin equilibrium; TAE, tannic acid equilibrium.

* *p* < 0.05.

** *p* < 0.001.

#### 3.2.4 Antioxidant Properties of FRB and NFRB

The antioxidant capacities of rice bran were changed by the fermentation process, as illustrated in Table 2. This study also directly confirmed that antioxidant activity is significantly (*p* < 0.05) higher in fermented rice bran (FRB) across multiple metrics like total phenolic content (TPC), total flavonoid content (TFC), and tannin. The elevated TPC positively correlated with enhanced free radical scavenging activity, as validated by the DPPH assays, and is considered a robust in vitro model (Mansouri et al. 2005). Structurally, phenols comprise an aromatic ring bearing one or more hydroxyl substituents. The antioxidant activity of this type of molecule is due to its ability to scavenge free radicals, donate hydrogen atoms or electrons, or chelate metal cations (Amarowicz et al. 2004). FRB extracts exhibited substantially lower IC_50_ (2.38 mg/mL) than NFRB (3.17 mg/mL), indicating superior potency for quenching the DPPH radical due to higher antioxidant phytochemicals post‐fermentation. Furthermore, FRB has a total antioxidant activity that is 21.6% more than NFRB's. Overall, fermentation demonstrates the potential for developing antioxidant‐enriched rice bran.

#### 3.2.5 Fatty Acid Profile

The gas chromatography (GC) with FID analysis characterized the fatty acid composition of FRB (fermented rice bran) and NFRB (nonfermented rice bran), as described in Table 3. Approximately 13 known excellent sources of fatty acids have been identified, accounting for over 99% of the total composition. However, more investigation is required for the remaining (0.39% to 0.40%) unknown compounds. The most abundant fatty acids found in FRB were monounsaturated and polyunsaturated varieties. Specifically, oleic acid and linoleic acid were present in high amounts. Research consistently shows that replacing saturated fats with unsaturated fats, particularly polyunsaturated fats, can lower LDL cholesterol and reduce the risk of coronary heart disease (DiNicolantonio and O'Keefe 2018; Sacks and Katan 2002). The fermentation process also reduced the amounts of saturated fats in FRB, like palmitic and stearic acid, compared to NFRB. Greater unsaturated‐to‐saturated fatty acid ratios are considered beneficial for health. The increased presence of monounsaturated fatty acids like oleic acid and polyunsaturated fatty acids like linoleic acid makes fermented rice bran become a functional food. These compounds have demonstrated positive impacts on markers of metabolic health. Recent studies have consistently shown that unsaturated fats, particularly monounsaturated and polyunsaturated fatty acids, can improve insulin sensitivity and blood glucose regulation in individuals with type 2 diabetes (Cardoso et al. 2021; Thota et al. 2019). The high unsaturated fat content of FRB may thus help manage type 2 diabetes. Research suggests that unsaturated fats, particularly omega‐3 and omega‐6 polyunsaturated fatty acids, have anti‐inflammatory properties that can mitigate the cardiovascular risks associated with diabetes (Tortosa‐Caparrós et al. 2017). The rise in unsaturated fats and decline in saturated fats offer health advantages, particularly beneficial for individuals with type 2 diabetes.

**TABLE 3:** Fatty acid composition of rice bran before and after fermentation.

List of fatty acids	Rice bran types	
Saturated	NFRB (%)	FRB (%)
C14:0 Myristic acid	0.38	0.38
C16:0 Palmitic acid	20.11	19.31
C17:0 Heptadecanoic acid	0.03	0.01
C18:0 Stearic acid	2.57	2.48
C 20:0 Arachidic acid	0.96	1.03
C 22:0 Behenic acid	0.28	0.33
C 24:0 Lignoceric acid	0.44	0.54
Total saturated	24.77	24.07
**Monounsaturated**		
C16:1 Palmitoleic acid	0.22	0.20
C18:1 cis‐9‐Oleic acid	42.32	44.31
C18:1 cis‐11‐Vaccenic acid	1.34	1.45
C 20:1 cis‐11‐eicosanoic acid	0.45	0.48
Total Monounsaturated	44.33	46.44
**Polyunsaturated**		
C 18:2 Linoleic acid	29.22	27.76
C 18:3 Alpha linolenic acid	1.32	1.34
Total polyunsaturated	30.54	29.10
Unknown	0.38	0.40

Abbreviations: FRB, fermented rice bran; NFRB, nonfermented rice bran.

#### 3.2.6 Organic Contents Analysis Using GC–MS

The GC–MS chromatogram (Figure S2) of the hexane extract of FRB (fermented rice bran) and NFRB (nonfermented rice bran) revealed the presence of 36 and 39 organic compounds, respectively. These compounds were characterized by their retention time (RT) and concentrations, according to the peak area, and are presented in Table S2. In this study, GC–MS results indicated that fermented rice bran is a richer source of n‐hexadecanoic acid than nonfermented rice bran (peak area 22.76% in FRB and peak area 16.24% in NFRB), and it has been reported that n‐hexadecanoic acid possesses some biological activity, such as antioxidant, hypocholesterolemic, anti‐inflammatory, and anti‐cancer (Fernandes et al. 2013; Harada et al. 2002; Korbecki and Bajdak‐Rusinek 2019). Additionally, 6‐octadecanoic acid and Hexadecanoic acid butyl ester were found in FRB, and these compounds have antimicrobial activity (Fernandes et al. 2013).

From the nutritive trait's evaluation of the FRB and NFRB perspectives, FRB is more beneficial for human health. Our results suggest FRB may be used as functional food ingredients or nutraceuticals and dietary supplements for various lifestyle‐related disorders of human beings.

### 3.3 Chronic Diet Supplementation: Assessment of Food and Water Intake, Fecal Excretion, Body Weight, and Cognitive Function

To assess the general physiological effects of fermented rice bran (FRB) compared to nonfermented rice bran (NFRB), we experimented with four different rat groups. After the acclimatization period, the ND group received a normal diet, while the other three groups received a high‐fat diet (HFD), HFD with 20% fermented rice bran (FRB group), and HFD with 20% nonfermented rice bran (NFRB group). All groups had free access to diet and fresh water for 8 weeks. Our findings from this study shed light on the potential benefits of FRB in improving physiological health status and could have significant implications for the future of nutrition research.

**FIGURE 1:**
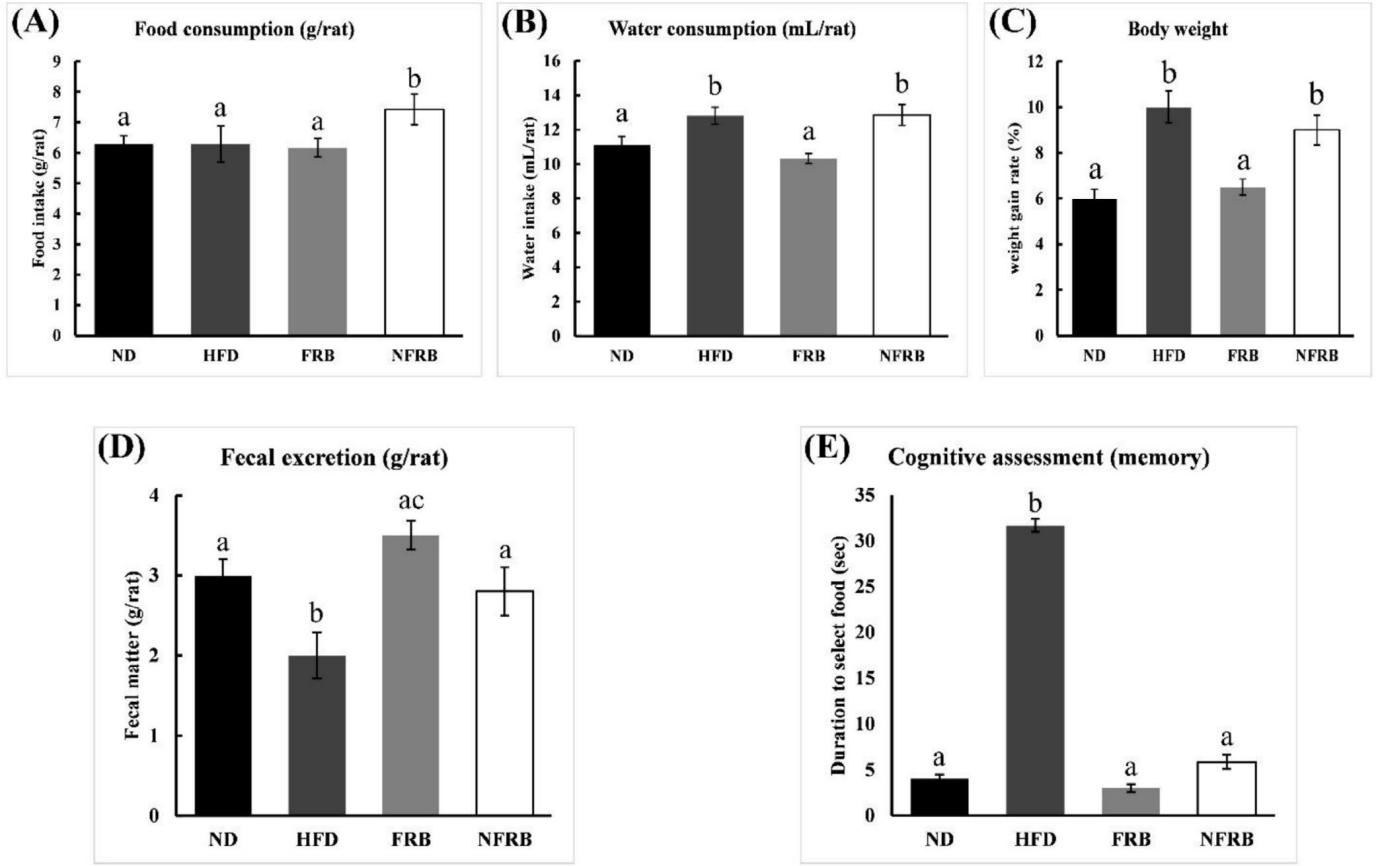
(A) Daily food intake (g) of rats, (B) daily water intake (mL), (C) weight gain rate as a percentage, (D) fecal excretion (g) of each rat, and (E) cognitive function (memory) of rats in each group (*n* = 6 per group). ND, normal diet; HFD, high‐fat diet; FRB, fermented rice bran; NFRB, nonfermented rice bran. Data are presented as mean ± SD. Statistical analysis was performed using one‐way ANOVA, followed by Tukey's HSD post hoc test (GraphPad Prism, version 10). Statistical significance was considered at *p* < 0.05. Different lowercase letters (a, b, c, d) indicate statistically significant differences among groups.

Figure 1A,B illustrate the average food and water intake of control and experimental animals after chronic supplementation of the diet. According to the results, there was no significant difference in food intake (*p* > 0.05) between the groups (except NFRB). However, the NFRB group consumed significantly more food than the FRB group (*p* = 0.03). Previous studies show that chronic intake of a high‐fat diet induces changes in plasma ghrelin and leptin levels, leading to relative acceleration of gastric emptying and is linked to increased energy intake and body weight, playing a significant role in the pathogenesis of obesity (Handjieva‐Darlenska and Boyadjieva 2009; Heshka and Jones 2001; Little et al. 2007). Our results indicate that FRB could have more impact than NFRB on regulating leptin stimulation, the hormone that helps feel full and satisfied. However, there is no significant difference found between the ND and FRB groups, which means similar satiety levels between the animals of both groups.

During the acclimatization period, all animals took the same amount of water on average. However, after grouping, at the beginning of the modified diet supplementation, it was noticed that the animals of the HFD group consumed more water compared to the others, as described in Figure 1B. Moreover, there is a similarity in water consumption patterns between fat‐enriched foods such as FRB and NFRB, both of which exceed ND. The findings were logical, as the animal consuming a high‐fat diet naturally exhibited increased water consumption. It is important to note that the consumption of a high‐fat diet (HFD) was gradually lower than that of the other groups, which may occur due to the delayed digestion typically associated with HFD (Li et al. 2011).

Eight weeks of supplementation with a normal and modified diet significantly changes body weight and impacts fecal excretion in the albino rat model. Figure 1C,D illustrate the average body weight gain rate (in percentage) and daily fecal discharge (per mice in grams) of all groups after chronic supplementation of the diet. The results demonstrate that the FRB diet effectively reduced body weight compared to HFD and NFRB. However, no significant difference was found between the FRB and ND groups in terms of body weight gain. These results indicate that FRB has a definite weight‐lowering effect. Interestingly, animals in the HFD group gained the highest weight (more than 10%) among groups, but exhibited poor fecal discharge rates. A previous study also shows that a high‐fat diet is associated with weight gain and constipation (Little et al. 2007; Mukai et al. 2020). In the aforementioned Figure 1A, food intake by the ND, HFD, and FRB group animals was quite similar. However, in the case of fecal excretion, the FRB group animals' fecal discharge rate was higher than that of HFD group animals. A previous study showed that FRB supplementation in mice fed a high‐fat diet for 8 weeks resulted in suppressed weight gain and significant alterations in the intestinal microbiota, indicating a potential role in mitigating obesity through gut microbiota modulation (Tochitani et al. 2022). Another study conducted in obese women concluded that wheat bran supplementation significantly reduced body weight and BMI (Elmadbouly 2015). During our elemental study of rice bran, it was observed that the fiber content of rice bran was increased after fermentation (Table 1). Possibly, the presence of more fiber in the FRB improves digestion and leads to reduced weight gain, constipation, and overall promotes gut health by gut microbiota modulation.

Figure 1E represents the result of the intelligence test, which was conducted through the diet‐choosing duration assessment on the control and experimental animals. After conducting multiple comparisons among groups using post hoc (Tukey's HSD) analysis, the findings revealed that the FRB group exhibited superior cognitive function compared to the HFD group (*p* < 0.0001), in terms of food detection within a shorter period. Despite the FRB group's high‐fat diet, they demonstrated a quicker ability to detect their meals. Additionally, the HFD, FRB, and NFRB groups are significantly (*p* < 0.05) different compared to the ND group. Previous studies reported the impaired learning and memory task consequences of chronic high‐fat diet administration (Sharma 2021; Winocur and Greenwood 2005). In this study, the optimized fermentation technique was used to produce the fermented rice bran enriched with several amino acids, most of them essential (Table 1). Prior studies state that dietary amino acid intake is associated with improved attention and cognitive function (Maruyamaand and Suzuki 1982). Tyrosine, an amino acid derived from phenylalanine, acts as a precursor to dopamine and norepinephrine, whereas tryptophan is a precursor to serotonin. These amino acids are important neurotransmitters for mood and attention regulation by boosting dopamine and serotonin levels. Research suggests that tyrosine and tryptophan supplementation is linked to enhanced cognitive performance and stress reduction (Aquili 2020; Bloemendaal et al. 2018). On the other hand, healthy fats, including monounsaturated and polyunsaturated fats, can improve brain health and cognition and are necessary for neurotransmitter production and myelin sheath insulation (Riviere et al. 2021; Shen et al. 2022). Our produced FRB is enriched with tyrosine, phenylalanine, and healthy fatty acids, as well as contains a marginal level of tryptophan. Another study investigated the effects of 20% stabilized rice bran supplementation on a rat model, showing a positive impact on glycemic control without adverse effects (Lee et al. 2014). This study supports that 20% FRB administration is safe and effective. So, choosing FRB as a dietary supplement may be an effective way to enhance cognitive function in people with intellectual disability in the upcoming era, which needs to be proved with clinical human trial data.

### 3.4 Chronic Diet Supplementation: Glucose Level and Lipid Profile

**FIGURE 2:**
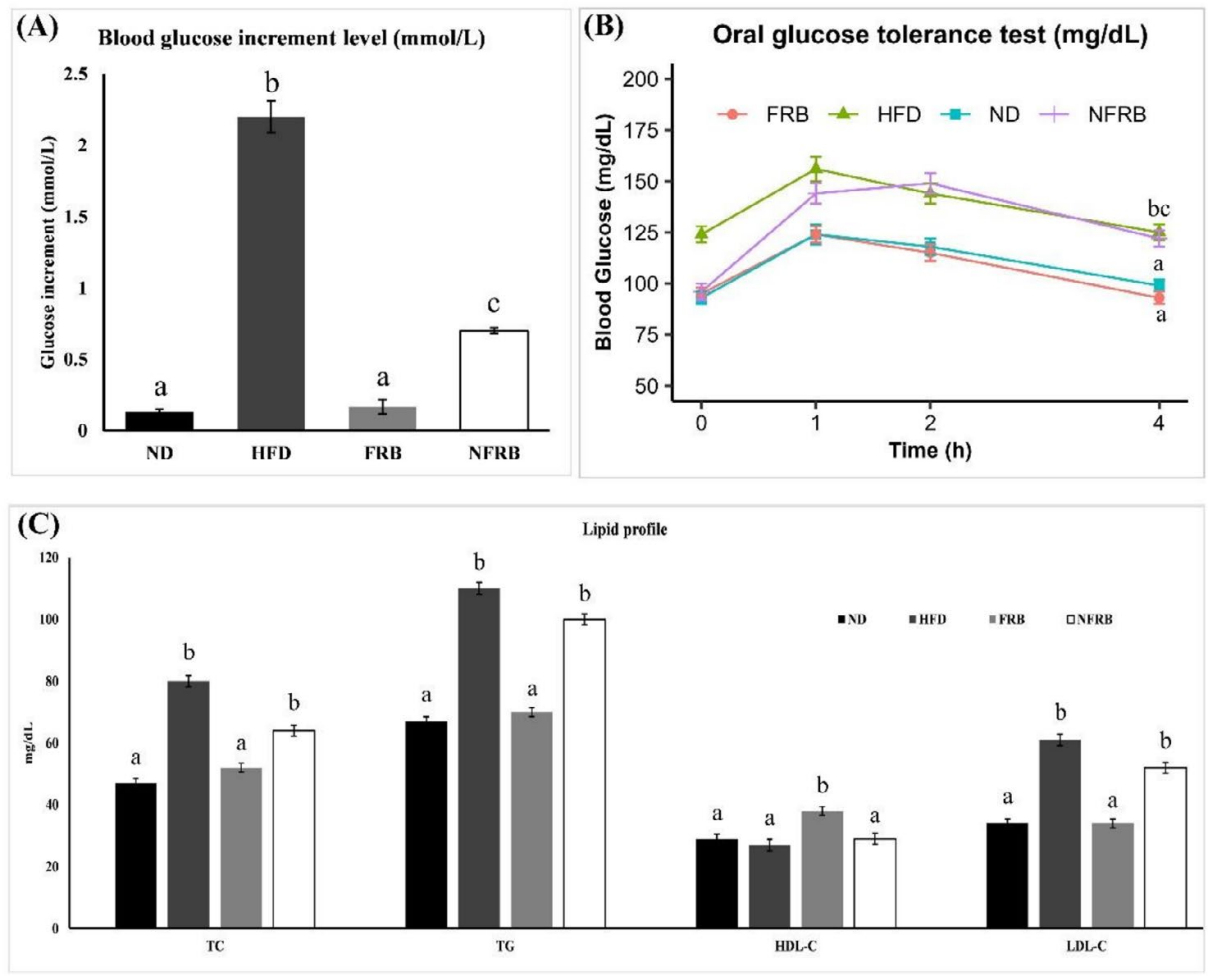
Effects of a normal diet and a modified diet on the blood glucose level and lipid profile of the rats at the end of the study. (A) represents the blood glucose increment level (mmol/L) in four groups of animals, whereas (B) states the blood glucose level (mg/dL) before (0 h) and after 1, 2, and 4 h of the OGTT test. (C) shows the lipid profile (TC, TG, HDL‐C, and LDL‐C) of all groups' animals as mg/dL units. Each group contains six rats (*n* = 6 per group). ND, normal diet; HFD, high‐fat diet; FRB, fermented rice bran; NFRB, nonfermented rice bran. Data are presented as mean ± SD. Statistical analysis was performed using one‐way ANOVA, followed by Tukey's HSD post hoc test (GraphPad Prism, version 10). Statistical significance was considered at *p* < 0.05. Different lowercase letters (a, b, c, d) indicate statistically significant differences among groups.

After 8 weeks of chronic normal and modified diet supplementation to the four groups of animals, we measured serum glucose levels (mmol/L) at overnight fasting conditions, as illustrated in Figure 2A. The results revealed significant variations (*p* < 0.05) in blood glucose levels among the different dietary groups. As expected, serum glucose levels of the HFD (high‐fat diet) group were significantly higher (*p* < 0.05) than those of the other groups. Interestingly, animals supplemented with Fermented Rice Bran (FRB) exhibited the lowest blood glucose levels among all experimental groups. Additionally, the NFRB (nonfermented rice bran) group was also able to moderately reduce the blood glucose levels. A meta‐analysis study based on an animal model showed that rice bran supplementation can significantly mitigate HbA1c (*p* = 0.003) level (Tantayakhom et al. 2025). Our findings and previous study results are well aligned and suggest that fermented rice bran has blood sugar‐lowering effects. So, FRB might be a source of nutraceuticals to combat metabolic syndrome.

Moreover, after 8 weeks, the oral glucose tolerance test (OGTT) was performed on each group of animals to evaluate glucose tolerance, as displayed in Figure 2B. According to the results, there were notable differences in each dietary group's ability to tolerate glucose. HFD and NFRB group animals were not able to maintain normal sugar levels in their blood after 2 h. Even after 4 h, their blood sugar level was diabetic (above 100 mg/dL). The ND group was able to tolerate high‐dose glucose supplementation. Interestingly, the FRB group was also able to maintain healthy sugar levels in their blood after oral glucose supplementation despite the FRB diet containing an 80% high‐fat diet. These findings reveal that a high‐fat diet is associated with glucose metabolism disorders, whereas fermented rice bran has the potential to mitigate these disorders. Several studies have established a clear link between a high‐fat diet and various health issues such as obesity, adiposity, insulin resistance, glucose intolerance, β‐cell dysfunction, and diabetes (Black et al. 2013; Daniel 2022; Tomar et al. 2023). Additionally, according to (Munkong et al. 2022), Red rice bran extract (RRBE) improves glucose‐insulin metabolism in mice as well as upregulates the expression of insulin, IDE, IRS, and GLUT genes. Furthermore, the fermentation process can improve the nutritional quality of rice bran through the synthesis of bioactive compounds, fiber, and unsaturated fatty acids. The literature review also supports that fermented foods and grain products can ameliorate glucose metabolism disorders and improve insulin resistance. On the basis of the literature review and our results, fermented rice bran has a potential therapeutic effect against people with glucose metabolism‐related disorders like diabetes.

At the end of the experiment, all groups of animals were sacrificed, and blood samples were collected to examine total cholesterol (TC), triglyceride (TG), high‐density lipoprotein cholesterol (HDL‐C), and low‐density lipoprotein cholesterol (LDL‐C) to evaluate the lipid profile, as illustrated in Figure 2C. The result reveals that the high‐fat diet receiving group of animals' TC level was significantly higher (*p* < 0.05) than the other groups. The NFRB group animal's TC level was slightly reduced compared to the HFD group; however, it was not significant. On the other hand, the fermented rice bran receiving group of animals was able to reduce TC levels in their blood to a normal level. Additionally, similar trends were observed in the case of TG and LDL‐C. In contrast, the good cholesterol or HDL‐C level was significantly higher (*p* < 0.05) in the FRB group compared to the other groups. Several previous studies showed that a high‐fat diet is associated with high triglyceride (TG), total cholesterol (TC), and low‐density lipoprotein cholesterol (LDL‐C) levels in mice and triggers atherosclerosis, steatosis, and depression‐like behavior (Kaprinay et al. 2016; Ruanpang et al. 2018). Furthermore, other studies reported that cereal bran and fiber consumption reduce blood pressure, total cholesterol, LDL cholesterol, and fasting blood glucose (Han et al. 2019; Zhu et al. 2023). A systematic review and meta‐analysis study revealed that rice bran supplementation improved lipid profiles, with reductions in total cholesterol and LDL cholesterol, and an increase in HDL cholesterol (Tantayakhom et al. 2025). So, fermented rice bran may be a good choice for patients with hyperlipidemia to improve their blood lipid profile, and ultimately might be a solution for metabolic syndrome.

### 3.5 Chronic Diet Supplementation: Liver Status and Hepatic Gene Expression

**FIGURE 3:**
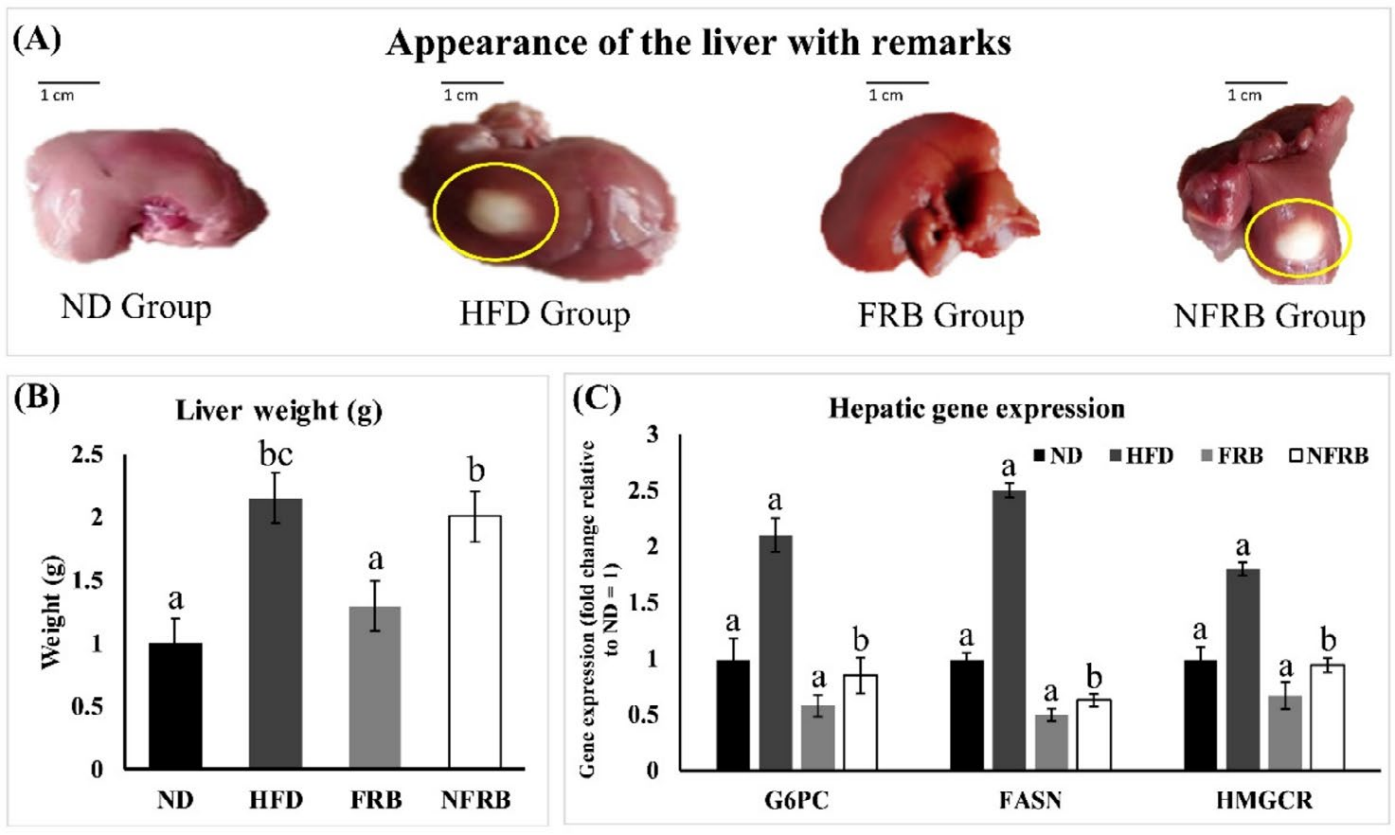
Effects of a normal diet and a modified diet on the liver appearance, weight, and hepatic gene expression of the rats at the end of the study. (3A) represents the appearance of the liver of all group animals with yellow oval remarks that indicate visible fat on the liver, scale bar 1 cm. (3B) shows the liver weight (g), and (3C) shows three different hepatic genes (G6PC, FASN, HMGCR) expressions in all group animals. Hepatic gene expression is presented as fold change relative to the ND group (set as 1). Each group contains six rats (*n* = 6 per group). G6PC, glucose‐6‐phosphatase catalytic subunit; FASN, fatty acid synthase; HMGCR, 3‐hydroxy‐3‐methylglutaryl‐CoA reductase. ND, normal diet; HFD, high‐fat diet; FRB, fermented rice bran; NFRB, nonfermented rice bran. Data are presented as mean ± SD. Statistical analysis was performed using one‐way ANOVA, followed by Tukey's HSD post hoc test (GraphPad Prism, version 10). Statistical significance was considered at *p* < 0.05. Different lowercase letters (a, b, c, d) indicate statistically significant differences among groups.

At the end of the study, all animals were sacrificed, and livers were collected to assess appearance, size, weight, and gene expression (Figure 3).

Figure 3A shows that HFD‐fed rats developed enlarged, fatty livers compared to the normal diet (ND) group. Consistently, liver weight in the HFD group was significantly higher (*p* < 0.05), exceeding 2 g (Figure 3B). In contrast, FRB supplementation normalized liver morphology, including appearance, size, and weight, with no visible fat deposition. NFRB also reduced hepatic fat accumulation, although a small residual fat layer was still observable. These findings align with previous reports demonstrating that HFD promotes fatty liver and contributes to metabolic dysfunction‐associated fatty liver disease (MAFLD) (Carmiel‐Haggai et al. 2005; Picchi et al. 2011), while rice bran derivatives can mitigate HFD‐induced hepatosteatosis and related metabolic disturbances (Munkong et al. 2022).

Figure 3C, an animal experiment was done to understand the detailed mechanism of the FRB effect on body weight gain and glucose‐lipid impairment of the high‐fat diet‐induced switch‐albino rat model. After 8 weeks of chronic supplementation of FRB, liver tissue was collected, and related genes (G6PC, FASN, and HMGCR) expression was analyzed. In the liver, there is de novo glucose synthesis mainly from lactate, alanine, pyruvate, and glycerol. In the present study, FRB reduced the mRNA levels of rate‐limiting enzymes involved in gluconeogenesis (G6PC), fatty acid synthesis (FASN), and lipogenesis (HMGCR). In this study, the aforementioned mechanisms were involved in the improvements in serum glucose and lipid levels by FRB. Therefore, chronic supplementation with FRB downregulated the mRNA expression levels of G6PC, FASN, and HMGCR in the liver (Figure 3C). A previous study reported that optimized rice bran extract (ORBE) suppressed high‐fat diet‐induced weight gain, hyperlipidemia, and hepatosteatosis in mouse models by activating AMPK and inhibiting STAT3, indicating its potential effectiveness in improving metabolic conditions related to lipid metabolism (Son et al. 2023). Thus, the decreased serum glucose, body weight, and liver TG, TC, and LDL‐c levels due to the intake of FRB may have been caused by the regulation of glucose and lipid metabolism. So, FRB can be a useful ingredient for encapsulated supplements to reduce the risk of metabolic disorders in humans.

### 3.6 Chronic Diet Supplementation: Liver Histology

**FIGURE 4:**
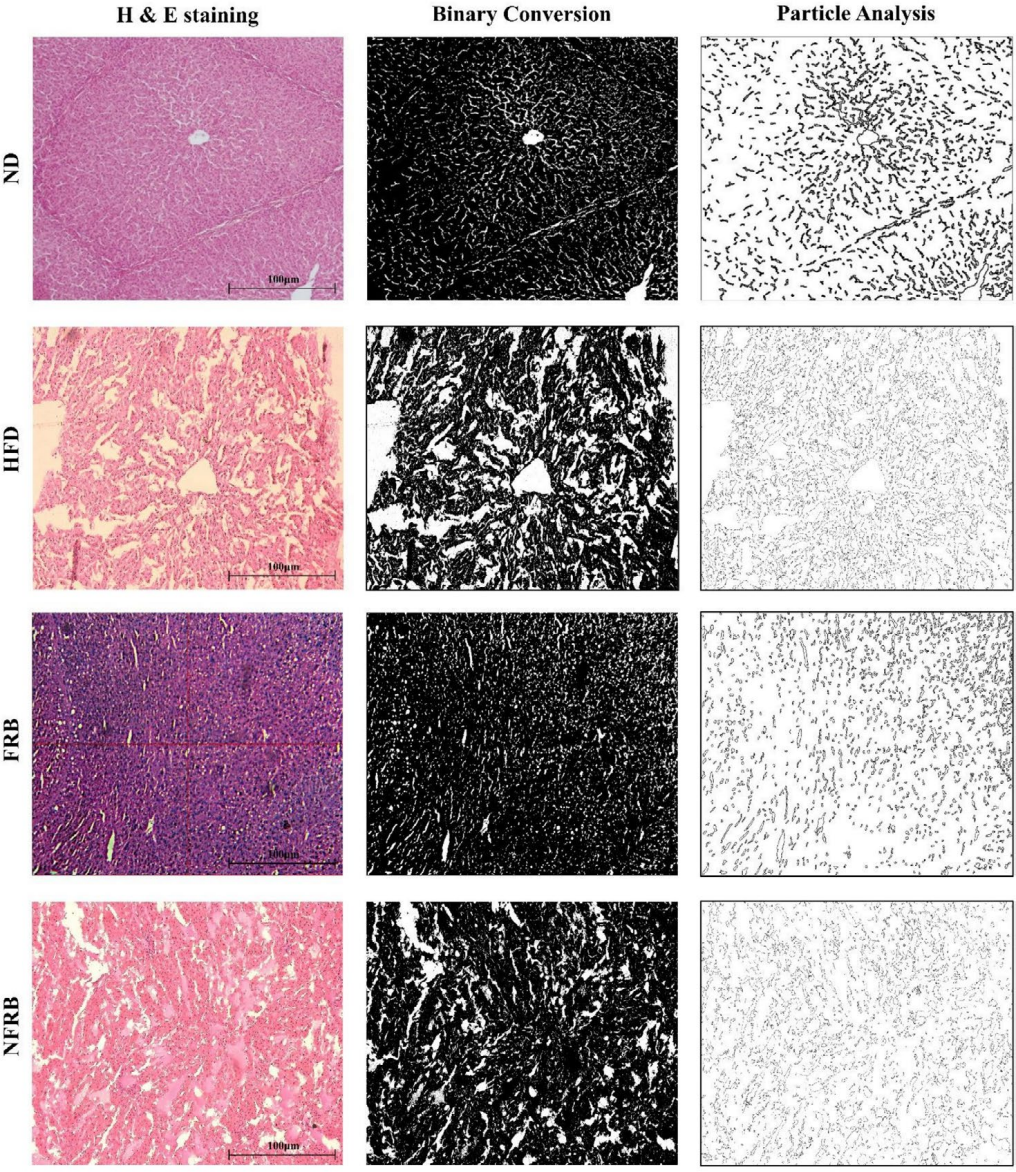
Effects of fermented rice bran on liver tissues. FRB, Fermented rice bran group; HFD, High‐fat diet group; ND, Normal diet group; NFRB, nonfermented rice bran group. Representative columns (from left to right) display the H&E staining of liver tissues (1st column), binary conversion (2nd column), and particle analysis (3rd column). Hematoxylin and Eosin (H&E) staining (200×, scale bar 100 μm and *n* = 6 per group).

We performed hepatic tissue staining to evaluate fermented and nonfermented rice bran on liver tissues, such as structural changes, fat accumulation, and necrosis. Figure 4 displays tissue staining of all the groups, where, from left to right, the 1st column is H&E staining, the 2nd column is binary conversion, and the 3rd column is particle analysis. High‐fat diet (HFD) receiving groups' liver tissue sections show structural changes, widened sinusoidal spaces, and hepatic necrosis. A previous study reported that a high‐fat diet causes enlarged sinusoidal spaces, disrupted hepatocytes, necrosis, and dilation of portal veins in hepatic sections (Asghar et al. 2021). Fat accumulation in hepatocytes can decrease blood flow through the liver's sinusoids, which are small capillaries. This physically compresses the sinusoids, causing them to narrow or even dilate in some areas. These structural changes can disrupt the normal flow of blood and oxygen within the liver, potentially contributing to liver injury or disease progression (Farrell et al. 2008; Nakadate et al. 2015).

Our findings suggest that the FRB (fermented rice bran) supplement improved liver tissue status, and the FRB group's tissue staining was almost similar to that of the normal diet‐receiving group (Figure 4). Studies reported that fermented foods can positively impact liver health, including reducing hepatic steatosis (fatty liver) and fat accumulation in liver tissues. These benefits are often linked to the probiotics and other beneficial bacteria and metabolites produced during fermentation, which can modulate gut health and reduce inflammation (Chen et al. 2016). Fermented foods or probiotics can modulate the gut microbiota through various mechanisms, including competition for colonization sites and nutrients, inhibition of pathogen growth through producing short‐chain fatty acids (SCFA), and antimicrobial agents produced by probiotics (Liu et al. 2022). Fermented foods contain compounds that can reduce inflammation, a key factor in the development and progression of MAFLD (Choi et al. 2024). Additionally, fermented foods can help regulate blood sugar levels, improve insulin sensitivity, and reduce the accumulation of triglycerides and cholesterol in the liver, all of which are beneficial for managing fatty liver disease (Jalili et al. 2023). Thus, our study findings align with previous study results, and we suggest fermented rice bran might have the potential to improve high‐fat diet‐induced liver status.

## 4 Conclusions

Nutritional composition and effect of fermented rice bran (FRB) derived from raw rice bran using optimized fermentation were investigated by conducting chemical analysis and an in vivo study. Proximate analysis and other analytical experiment results indicate that FRB has more nutritional value than NFRB (nonfermented rice bran). Fermented rice bran is rich in fiber and protein, as well as essential amino acids, fatty acids, phenolic contents, organic contents having beneficial biological activities, and antioxidant capacities. Moreover, an animal experiment revealed that FRB can reduce obesity in rats induced by a high‐fat diet and improve cognitive function (memory) without affecting daily food, water intake, or fecal excretion. Additionally, FRB supplements can maintain healthy blood glucose levels and lipid profiles and can combat fatty liver disease by improving liver tissues as well as downregulating the hepatic mRNA expression levels of G6PC, FASN, and HMGCR. These findings provide strong evidence that FRB supplementation may represent a functional dietary strategy to prevent obesity‐related metabolic dysfunction. This study was performed in six rats per group and lacked proven clinical human trial data. We suggest that further study is needed to prove the potential of fermented rice bran through a large group and a human trial.

## Author Contributions

**Afroza Sultana:** data curation (equal), formal analysis (equal), investigation (equal), methodology (equal), writing – original draft (equal), writing – review and editing (equal). **Md. Ruhul Amin:** data curation (equal), formal analysis (equal), methodology (equal), visualization (equal), writing – original draft (equal), writing – review and editing (equal). **Md. Omar Faruque:** supervision (equal), writing – review and editing (equal). **Muhammad Ali Siddiquee:** writing – review and editing (equal). **Md. Zakir Hossain Howlader:** writing – review and editing (equal). **Md. Alauddin:** conceptualization (equal), formal analysis (equal), funding acquisition (equal), investigation (equal), project administration (equal), supervision (equal), writing – review and editing (equal).

## Funding

This work was partially supported by the Jashore University of Science and Technology (grant no. 24‐FoAST‐06).

## Conflicts of Interest

The authors declare no conflicts of interest.

## Supporting information


**Figure S1:** Chromatograph of the NFRB (A) and FRB (B) amino acids.
**Figure S2:** GC–MS chromatogram of the FRB (A) and NFRB (B) organic compounds.

**Table S1:** Optimization of SPFT by assessing various parameters of FRB.
**Table S2:** Organic compounds identified in FRB and NFRB by GC–MS analysis.
**Table S3:** List of primers and respective sequences.

## Data Availability

Data will be available on request from the authors.
