# Supplementary material for: Fermented Rice Bran: A Promising Therapeutic Agent Against High‐Fat Diet‐Induced Metabolic Disorders
Source: Food Sci Nutr. 2026 Jan 9;14(1):e71439. doi: 10.1002/fsn3.71439 (PMC12789662; doi:10.1002/fsn3.71439)
Supplement: Supplementary file 1 — Figure S1: Chromatograph of the NFRB (A) and FRB (B) amino acids. Figure S2: GC–MS chromatogram of the FRB (A) and NFRB (B) organic compounds. [file FSN3-14-e71439-s001.docx]

**Supplementary figures**

**Figure S1**

**
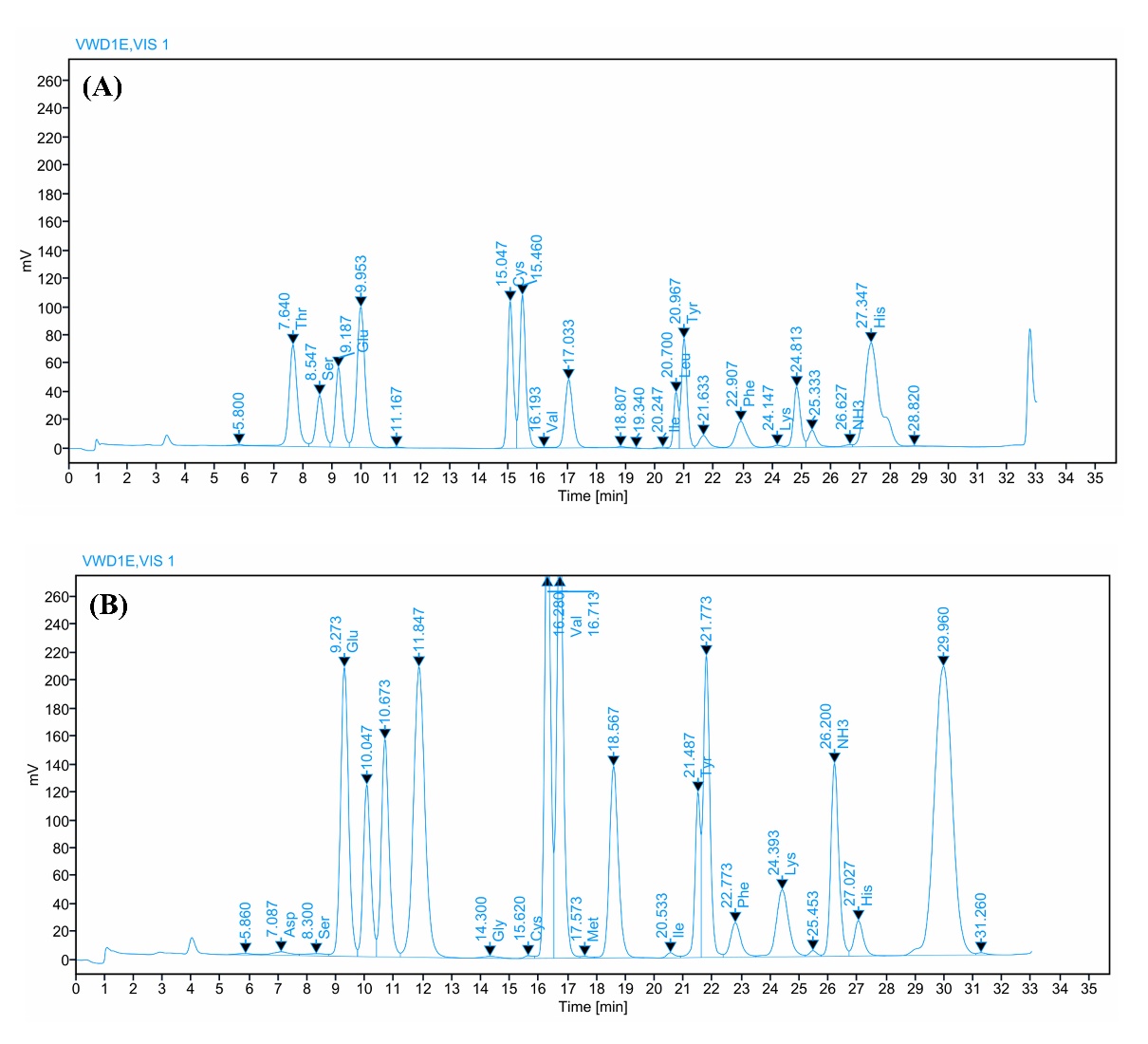
**

**Figure S1:** Chromatograph of the NFRB (A) and FRB (B) amino acids.

**Figure S2**


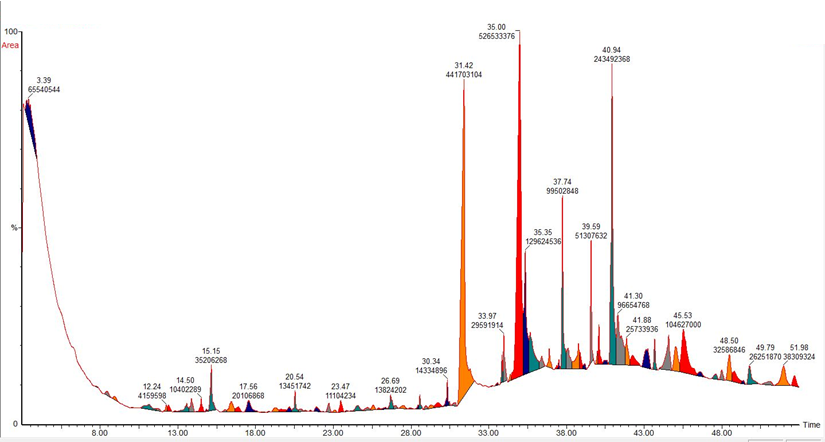

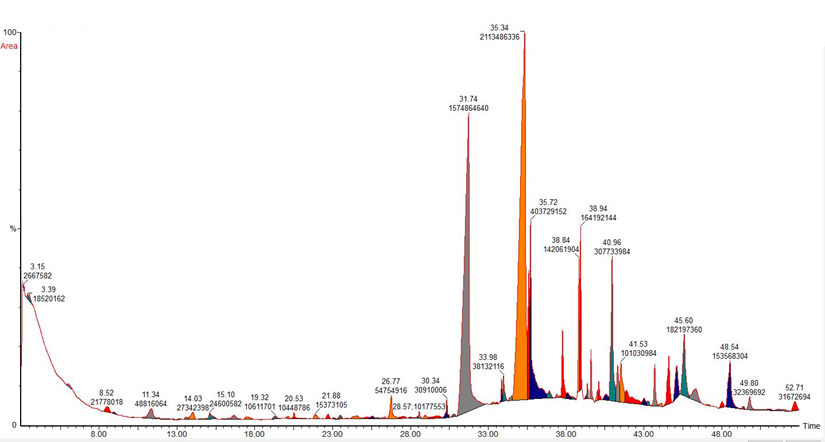


**(B)**

**(A)**

**Figure S2:** GC-MS chromatogram of the FRB (A) and NFRB (B) organic compounds.
