# Supplementary material for: Fermented Rice Bran: A Promising Therapeutic Agent Against High‐Fat Diet‐Induced Metabolic Disorders
Source: Food Sci Nutr. 2026 Jan 9;14(1):e71439. doi: 10.1002/fsn3.71439 (PMC12789662; doi:10.1002/fsn3.71439)
Supplement: Supplementary file 2 — Table S1: Optimization of SPFT by assessing various parameters of FRB. Table S2: Organic compounds identified in FRB and NFRB by GC–MS analysis. Table S3: List of primers and respective sequences. [file FSN3-14-e71439-s002.docx]

**Supplementary Table**

| **Table S1:** Optimization of SPFT by assessing various parameters of FRB. | | | | | |
| --- | --- | --- | --- | --- | --- |
| **Assessment parameters** | **SPFT** | | | | |
|  | Tech-1 | Tech-2 | Tech-3 | Tech-4 | Tech-5 |
| Phenolic Content (mg/g)^A^ | 206.0 | 100.0 | 90.0 | 150.0 | 95.0 |
| Fiber (%) | 22.0 | 18.0 | 19.0 | 21.0 | 20.0 |
| Fat (%) | 5.0 | 4.1 | 6.0 | 5.0 | 5.0 |
| Protein (%) | 12.6 | 10.0 | 9.5 | 11.0 | 10.0 |
| Moisture (%) | 4.0 | 4.0 | 4.9 | 5.5 | 5.0 |
| Pictorial view of fermented rice bran | 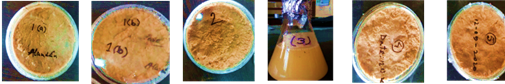 | 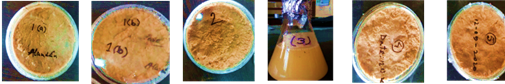 | 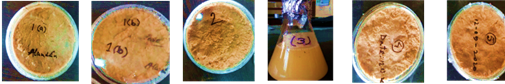 | 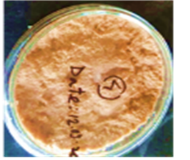 | 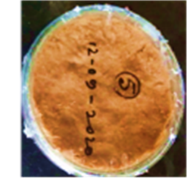 |
| SPFT, solid-phase fermentation technique; A) Phenolic Content expressed as mg of gallic acid equilibrium /g of dry extract of the sample. | | | | | |

| **Table S2: Organic compounds identified in FRB and NFRB by GC-MS analysis** | | | | | |
| --- | --- | --- | --- | --- | --- |
| **Compounds in FRB (Fermented Rice Bran)** | | | | | |
| **Identified Name** | **M.W.** | **Formula** | **RT (Min)** | **Peak Area (%)** | **Structure** |
| Heptanoic Acid | 130 | C_7_H_14_O_2_ | 8.506 | 0.31 | 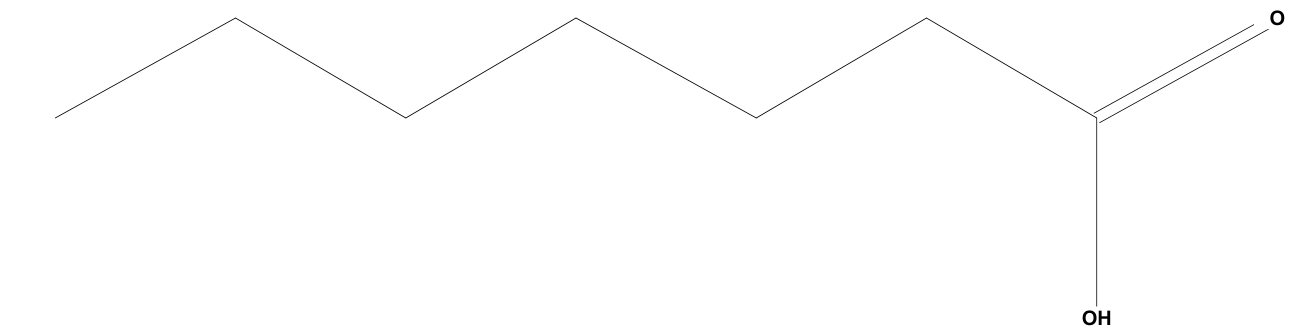 |
| Octanoic Acid | 144 | C_8_H_16_O_2_ | 11.332 | 0.7 | 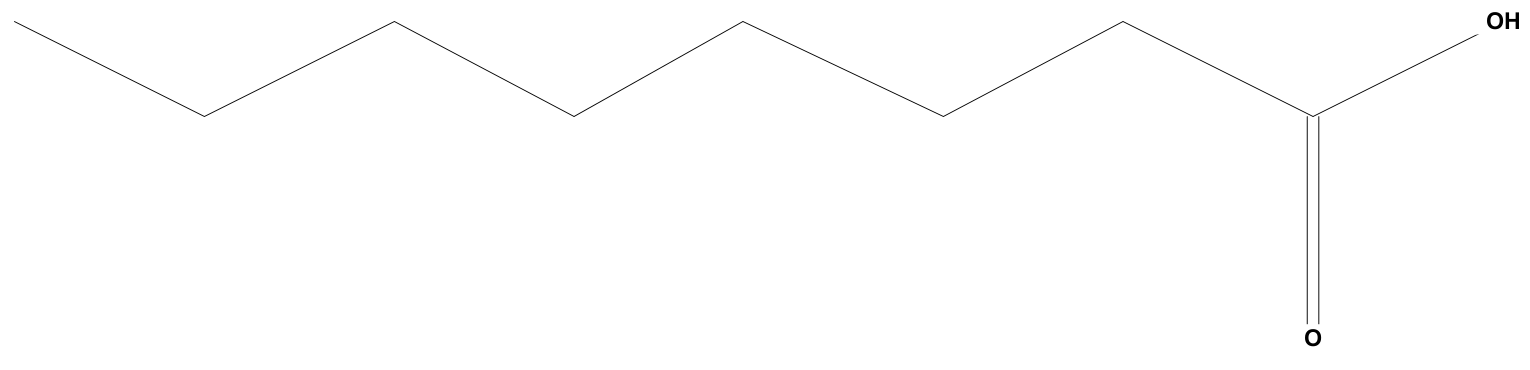 |
| N-Decanoic Acid | 172 | C_10_H_20_O_2_ | 14.039 | 0.4 | 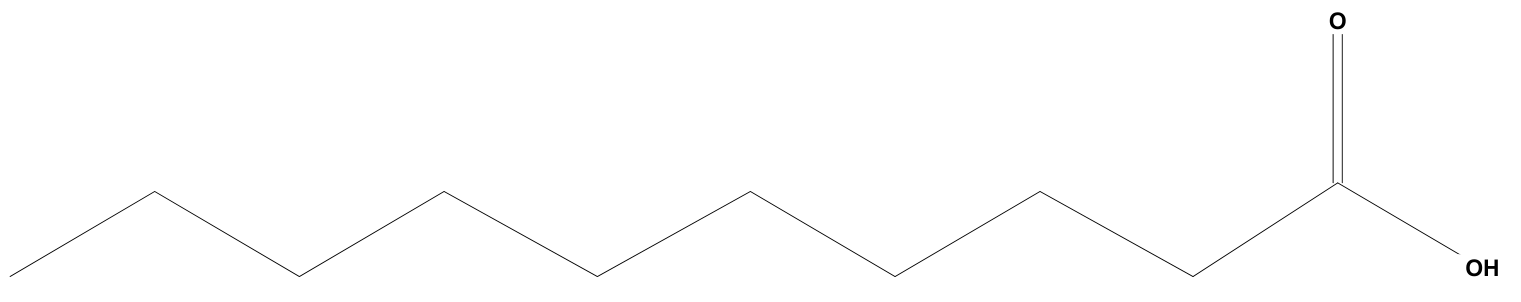 |
| Phenol, 3-Methyl-5-(1-Methylethyl)-, Methylcarbamate | 207 | C_12_H_17_O_2_N | 15.089 | 0.35 | 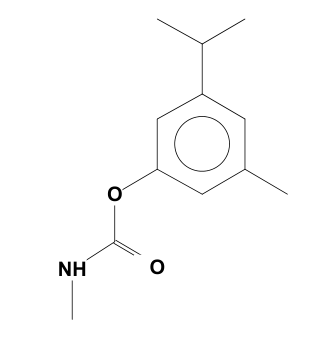 |
| N-Decanoic Acid | 172 | C_10_H_20_O_2_ | 16.67 | 0.32 | 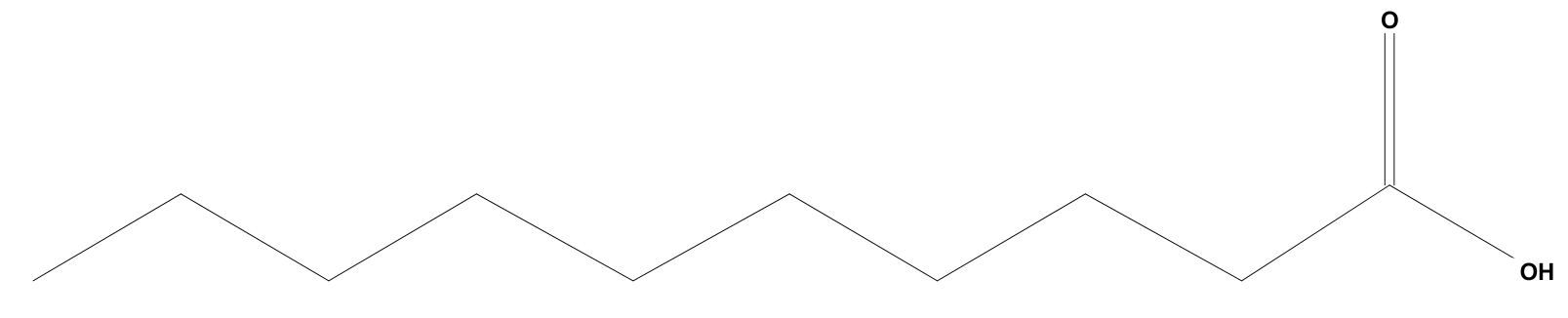 |
| N-Decanoic Acid | 172 | C_10_H_20_O_2_ | 19.32 | 0.15 |  |
| 2,4-Di-Tert-Butylphenol | 206 | C_14_H_22_O | 20.527 | 0.15 | 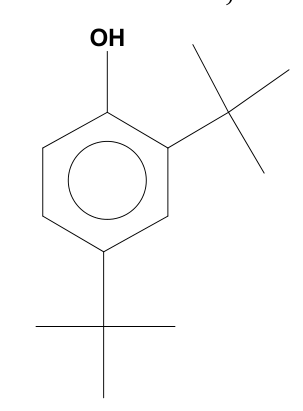 |
| Dodecanoic Acid | 200 | C_12_H_24_O_2_ | 21.88 | 0.22 | 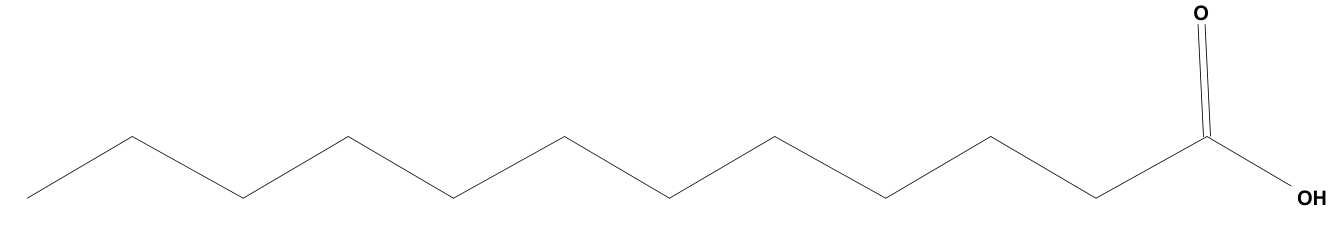 |
| Hentriacontane | 436 | C_31_H_64_ | 22.72 | 0.18 | 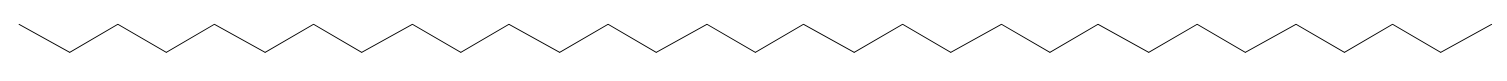 |
| Tetradecanoic Acid | 228 | C₁₄H₂₈O₂ | 26.764 | 0.79 | 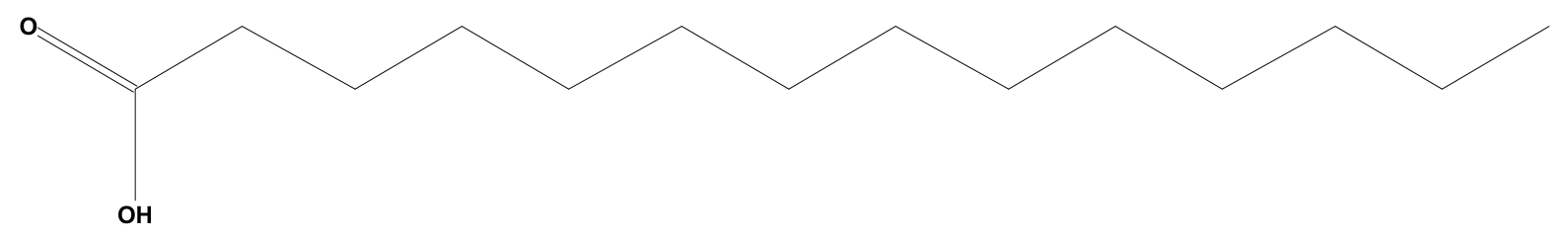 |
| 3-Methyl-2-(2-Oxopropyl) Furan | 138 | C₈H₁₀O₂ | 28.57 | 0.15 | 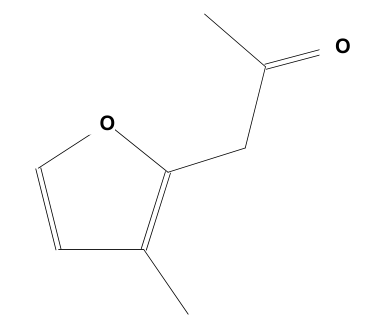 |
| Tetradecanoic Acid | 228 | C₁₄H₂₈O₂ | 29.95 | 0.3 | 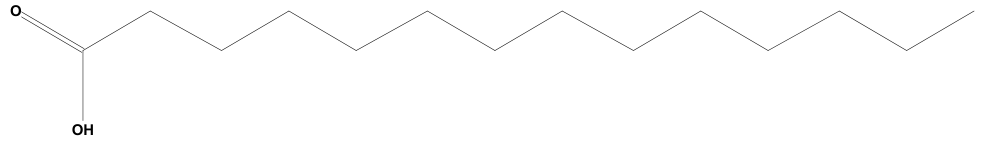 |
| Tetradecanoic Acid, 10,13-Dimethyl-, Methyl Ester | 270 | C₁₇H₃₄O₂ | 30.336 | 0.45 | 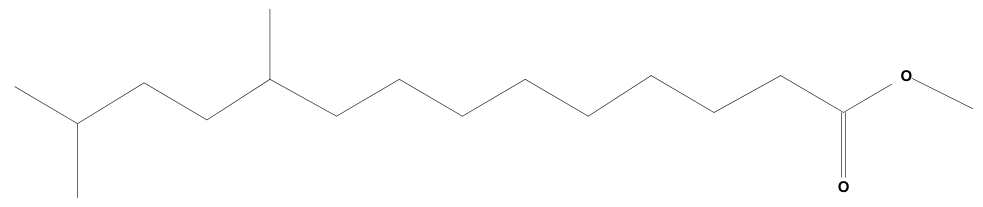 |
| N-Hexadecanoic Acid | 256 | C₁₆H₃₂O₂ | 31.747 | 22.76 | 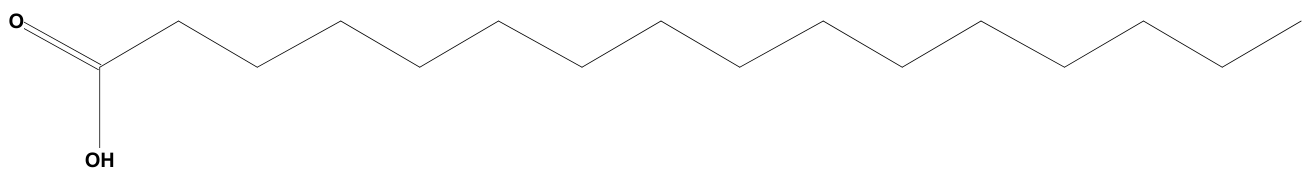 |
| 12,15-Octadecadienoic Acid, Methyl Ester | 294 | C₁₉H₃₄O₂ | 33.86 | 0.34 | 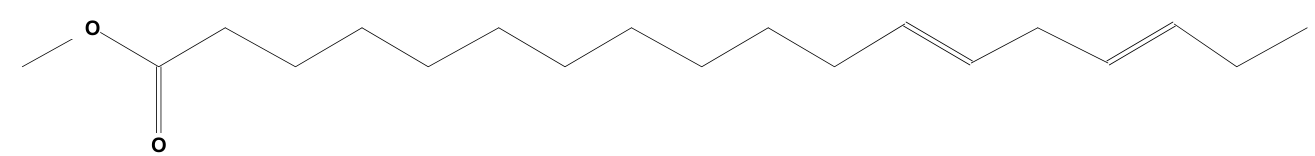 |
| 6-Octadecenoic Acid | 282 | C₁₈H₃₄O₂ | 33.98 | 0.55 | 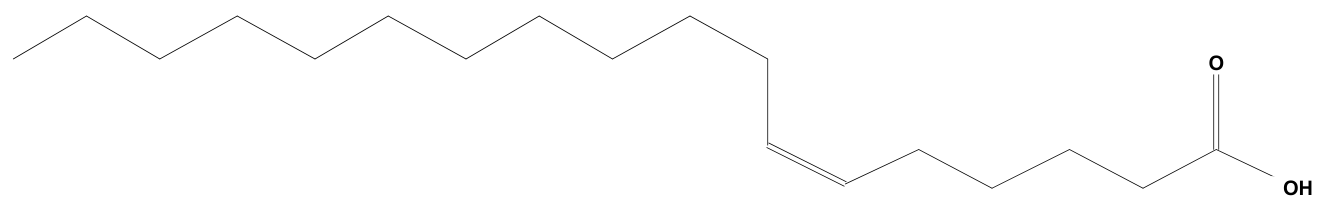 |
| 6-Octadecenoic Acid | 282 | C₁₈H₃₄O₂ | 35.34 | 30.55 |  |
| Octadecanoic Acid | 284 | C₁₈H₃₆O₂ | 35.62 | 3.11 | 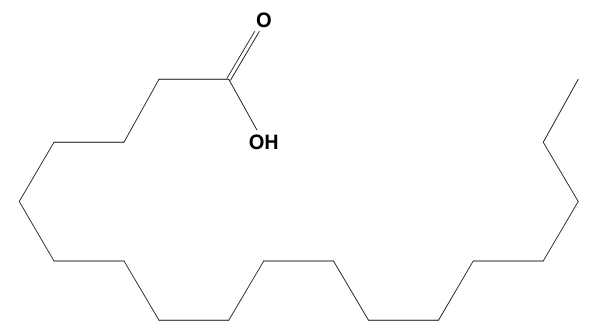 |
| Hexadecanoic Acid, Butyl Ester | 312 | C₂₀H₄₀O₂ | 35.72 | 5.84 | 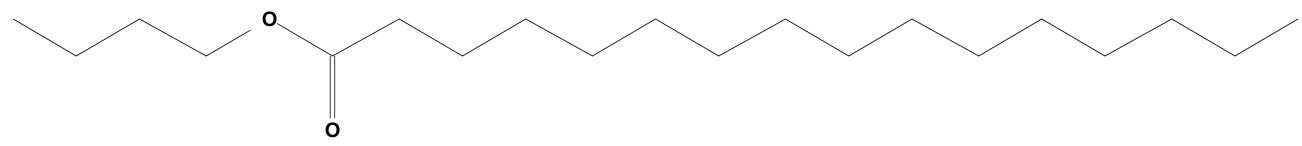 |
| Glycidyl Palmitate | 312 | C₁₉H₃₆O₃ | 37.77 | 1.62 | 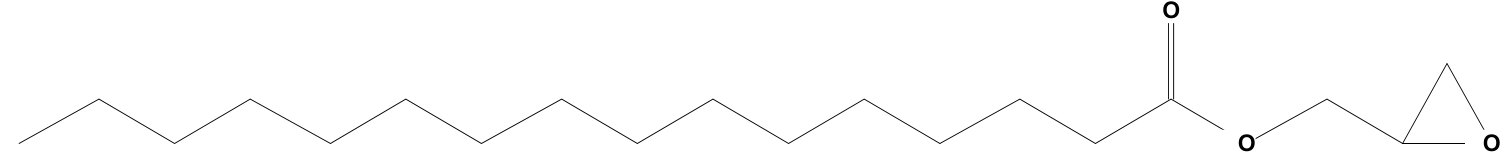 |
| Butyl 9,12-Octadecadienoate | 336 | C₂₂H₄₀O₂ | 38.84 | 2.05 | 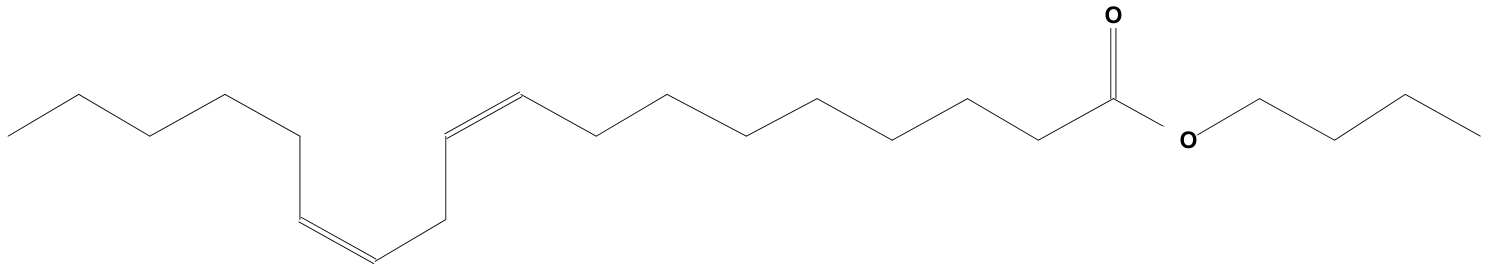 |
| 6-Octadecenoic Acid | 282 | C_18_H_34_O_2_ | 38.94 | 2.37 | 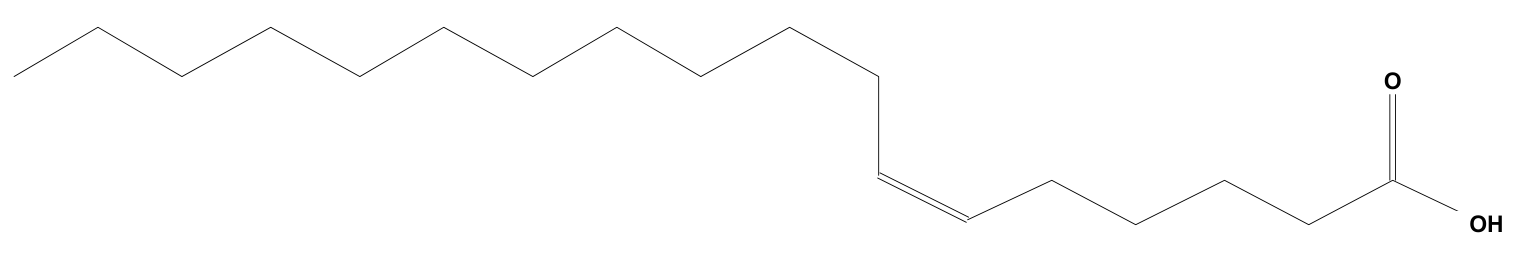 |
| Octadecanoic Acid, Butyl Ester | 340 | C₂₂H₄₄O₂ | 39.38 | 0.21 | 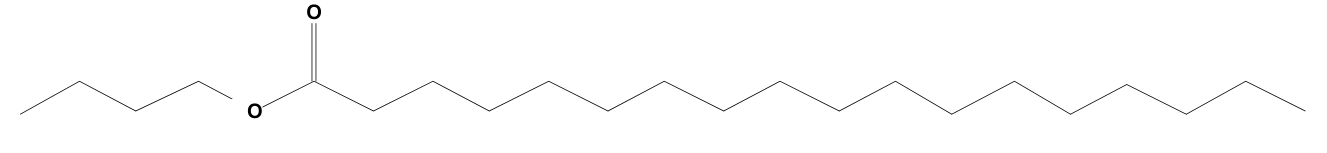 |
| Hexanedioic Acid, Bis(2-Ethylhexyl) Ester | 370 | C₂₂H₄₂O₄ | 39.61 | 0.72 | 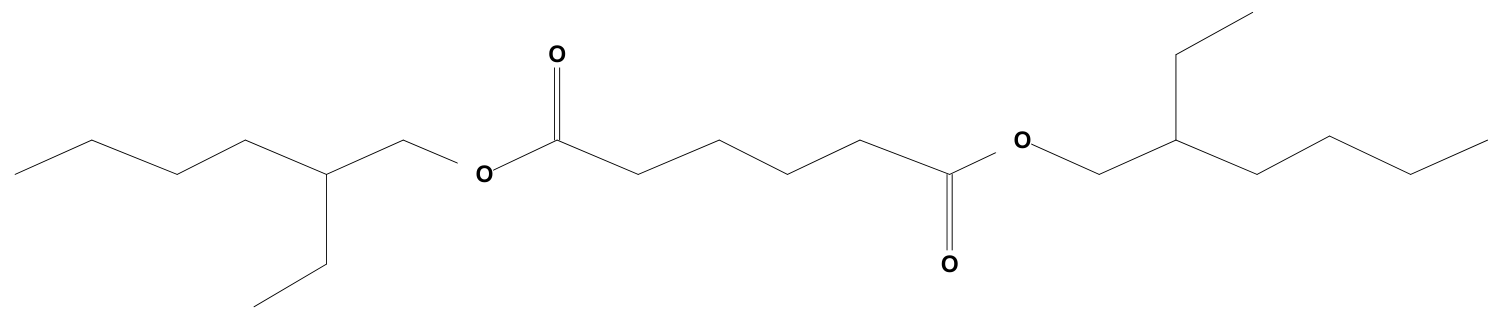 |
| 6-Octadecenoic Acid | 282 | C₁₈H₃₄O₂ | 40.11 | 0.36 | 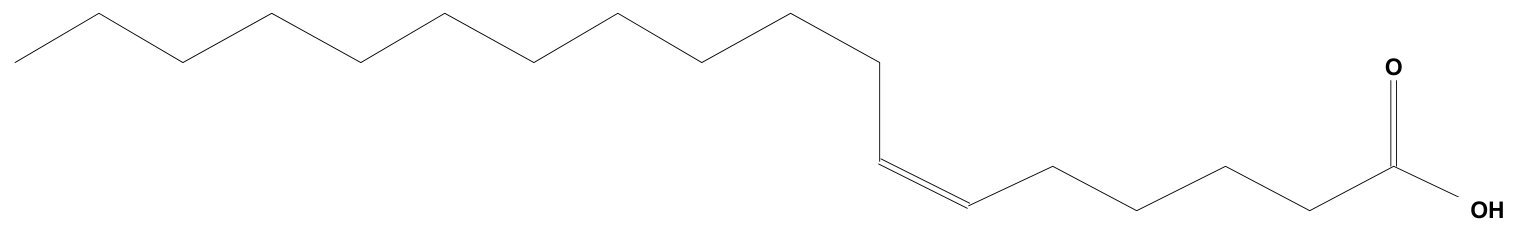 |
| Glycidyl Oleate | 338 | C₂₁H₃₈O₃ | 40.96 | 4.45 | 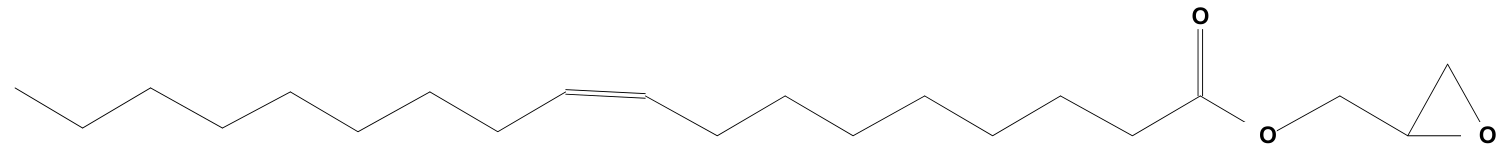 |
| Oxalic Acid, Monoamide, N-Allyl-, Hexadecyl Ester | 353 | C₂₁H₃₉O₃N | 41.32 | 1.13 | 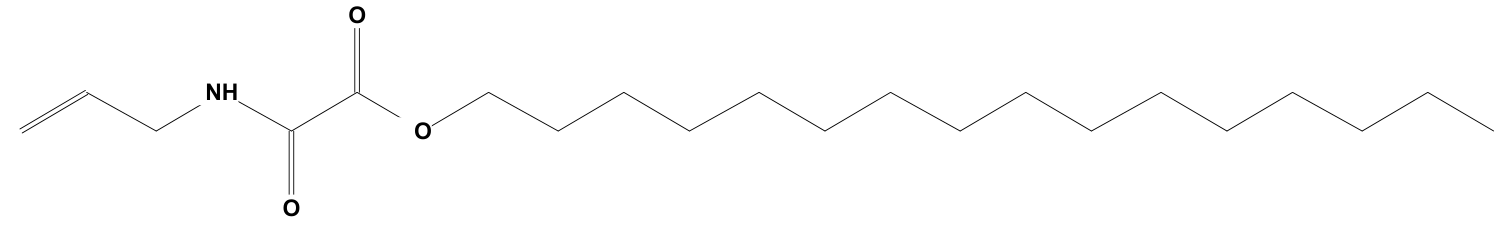 |
| 5-Methyl-Z-5-Docosene | 322 | C₂₃H₄₆ | 41.53 | 1.46 | 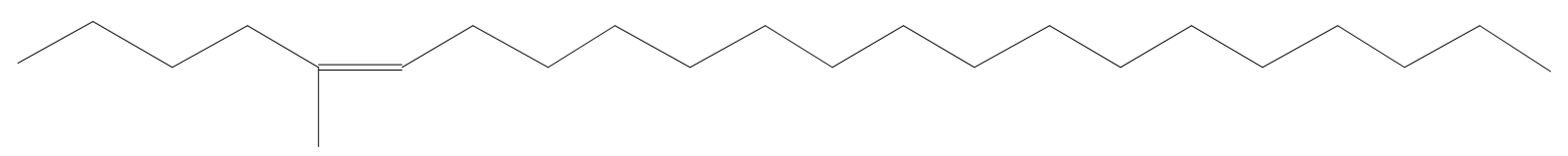 |
| Silane, Dimethyl (Dimethyl (But-3-Enyloxy) Silyloxy) Tridecyloxy- | 402 | C₂₁H₄₆O₃Si₂ | 43.7 | 0.99 | 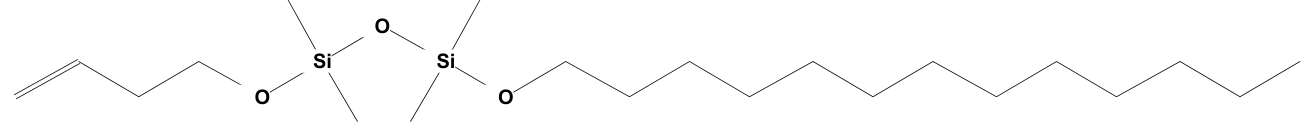 |
| 5,5-Dimethyl-1,3-Dioxane-2-Ethanol, Tbdms Derivative | 274 | C₁₄H₃₀O₃Si | 44.61 | 1.64 | 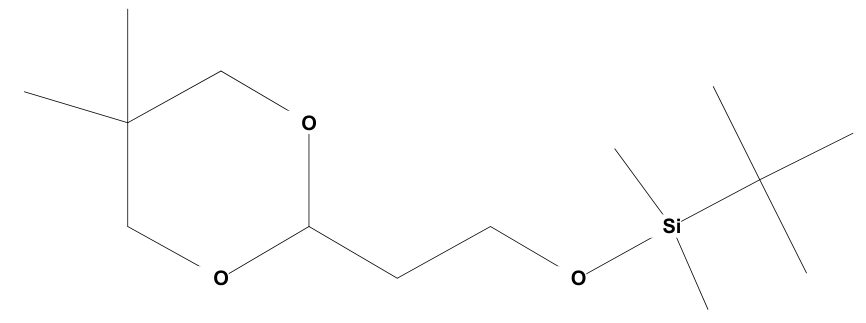 |
| Oleic Acid, (Z)-, Tms Derivative | 354 | C₂₁H₄₂O₂Si | 45.11 | 1.37 | 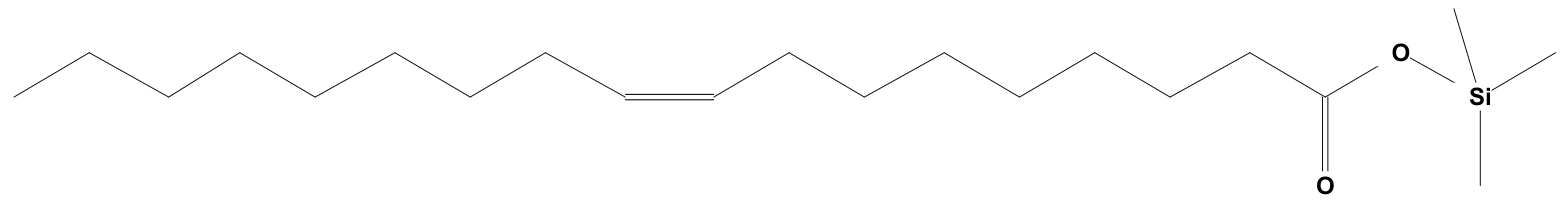 |
| 6-Octadecenoic Acid | 282 | C₁₈H₃₄O₂ | 45.6 | 2.63 | 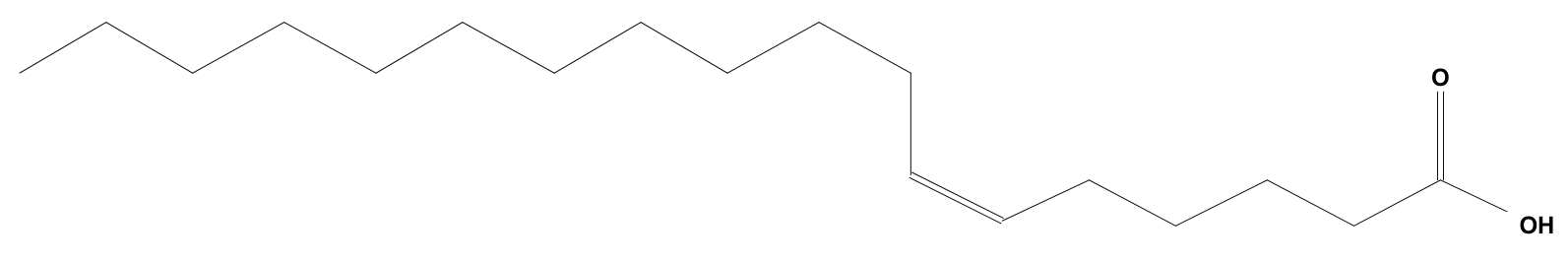 |
| I-Propyl 11,12-Methylene-Octadecanoate | 338 | C₂₂H₄₂O₂ | 48.03 | 0.23 | 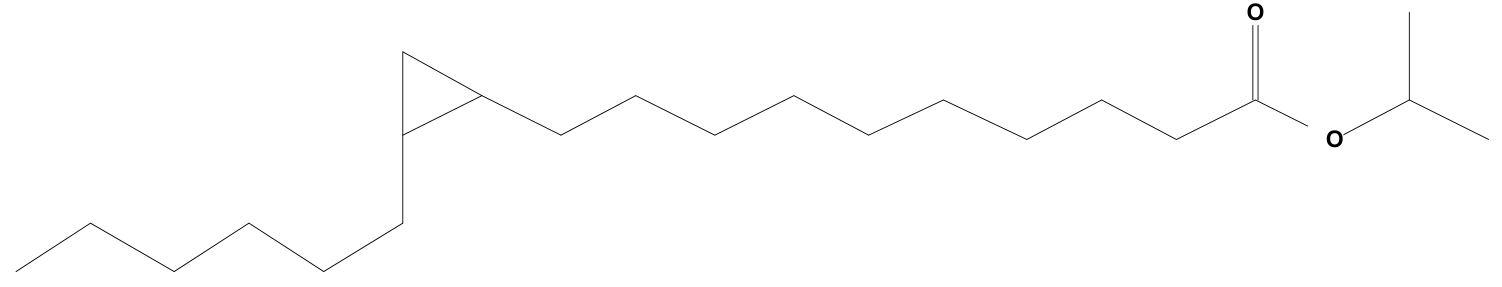 |
| Silane, Dimethyl (Dimethyl (But-3-Enyloxy) Silyloxy) Butoxy- | 276 | C₁₂H₂₈O₃Si₂ | 48.54 | 2.22 | 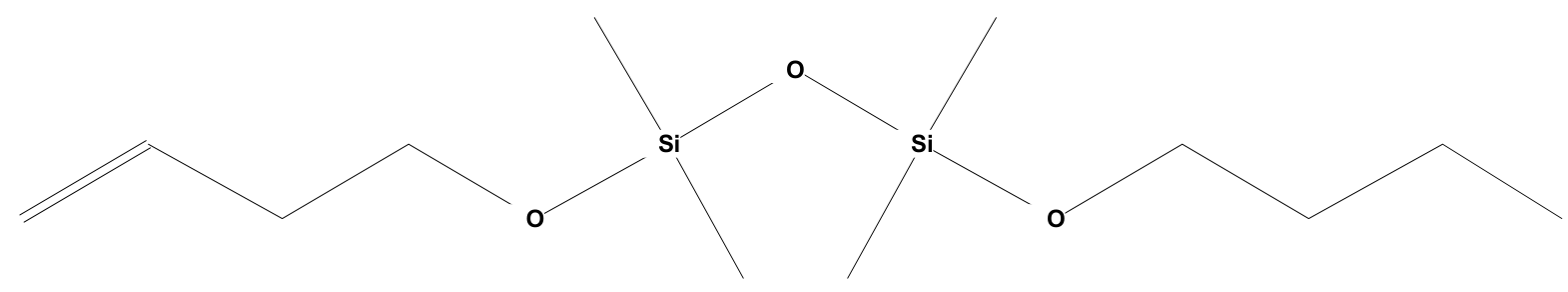 |
| Squalene | 410 | C_30_H_50_ | 49.8 | 0.47 | 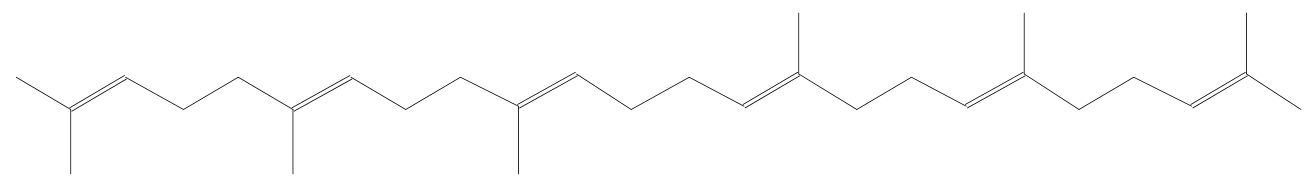 |
| Hentriacontane | 436 | C_31_H_64_ | 52.71 | 0.46 | 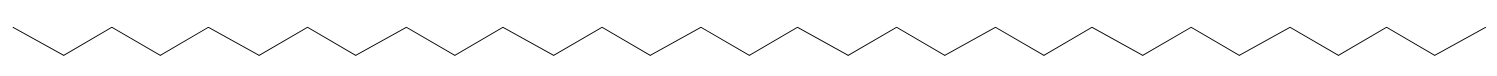 |
| **Compounds in NFRB (Non-Fermented Rice Bran)** | | | | | |
| Benzenemethanesulfonyl Fluoride | 174 | C₇H₇O₂FS | 12.24 | 0.15 | 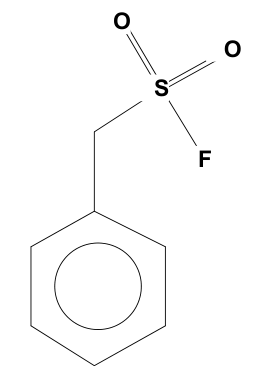 |
| 2-Decenal, (E)- | 154 | C₁₀H₁₈O | 13.56 | 0.46 | 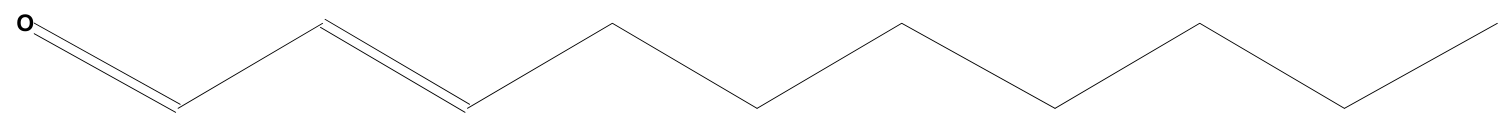 |
| Pyrido[2,3-D] Pyrimidine | 131 | C₇H₅N₃ | 13.86 | 0.46 | 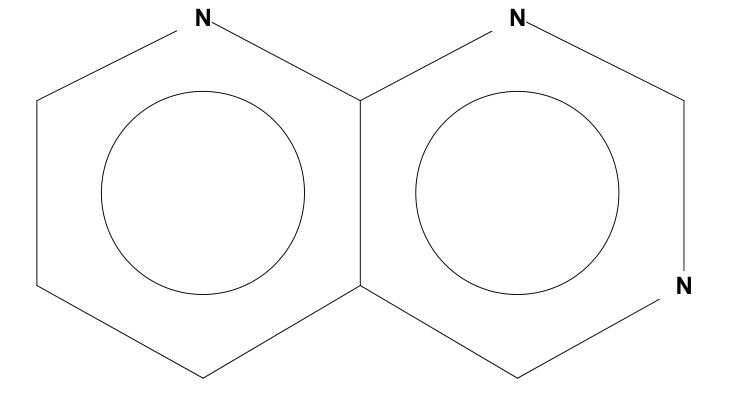 |
| 2,4-Decadienal, (E, E)- | 152 | C₁₀H₁₆O | 14.5 | 0.38 | 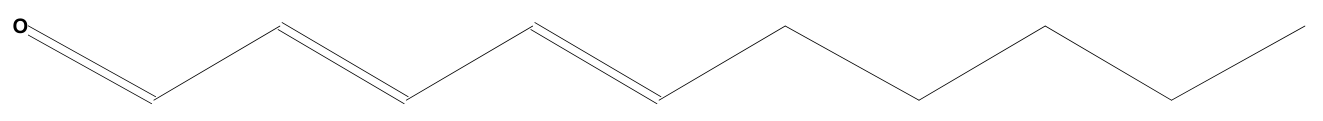 |
| 2,4-Nonadienal, (E, E)- | 152 | C₁₀H₁₆O | 15.15 | 1.29 | 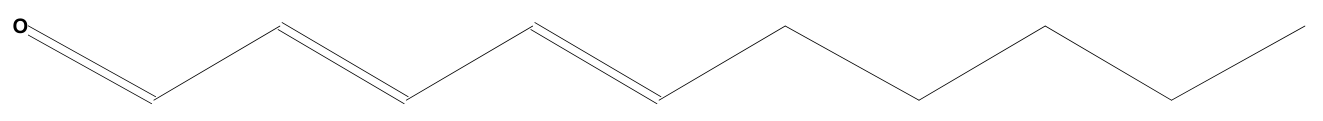 |
| 3-Methyl-2-(2-Oxopropyl) Furan | 138 | C₈H₁₀O₂ | 16.43 | 0.62 | 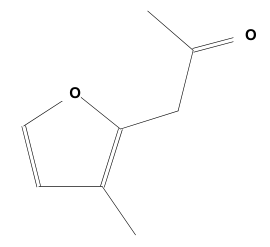 |
| 2-Cyclopenten-1-One, 4-Acetyl-2,3,4,5,5-Pentamethyl- | 194 | C₁₂H₁₈O₂ | 17.55 | 0.74 | 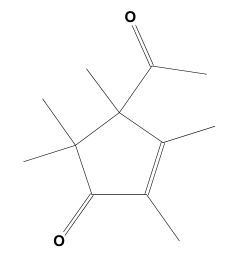 |
| Cycloheptasiloxane, Tetradecamethyl- | 518 | C₁₄H₄₂O₇Si₇ | 20.17 | 0.23 | 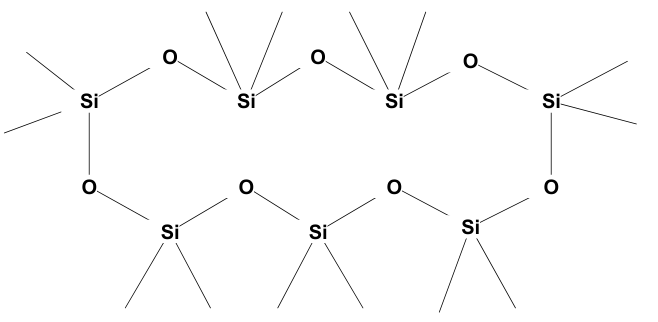 |
| 2,4-Di-Tert-Butylphenol | 206 | C₁₄H₂₂O | 20.54 | 0.5 | 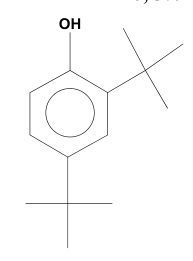 |
| 1-Methyl-3,6-Diazahomoadamantan-9-One | 180 | C₁₀H₁₆ON₂ | 21.9 | 0.27 | 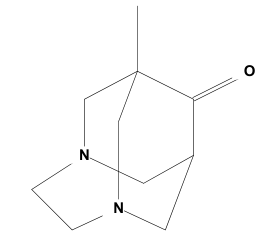 |
| Hentriacontane | 436 | C₃₁H₆₄ | 22.72 | 0.32 | 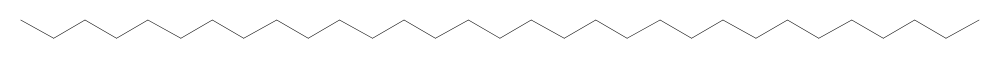 |
| 1,2-Benzisothiazol-3(2h)-One, 2-Methyl-, 1,1-Dioxide | 197 | C₈H₇O₃NS | 23.46 | 0.4 | 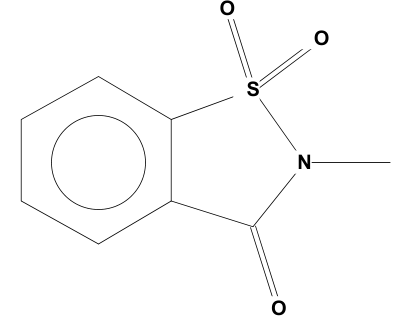 |
| 3-Methyl-2-(2-Oxopropyl) Furan | 138 | C₈H₁₀O₂ | 24.57 | 0.4 | 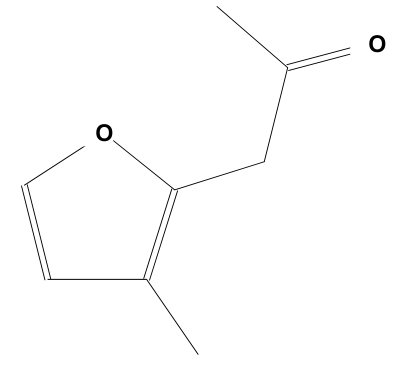 |
| Tetracontane-1,40-Diol | 594 | C₄₀H₈₂O₂ | 25.55 | 0.2 | 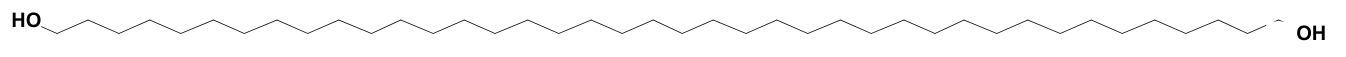 |
| Tetradecanoic Acid | 228 | C₁₄H₂₈O₂ | 26.69 | 0.5 | 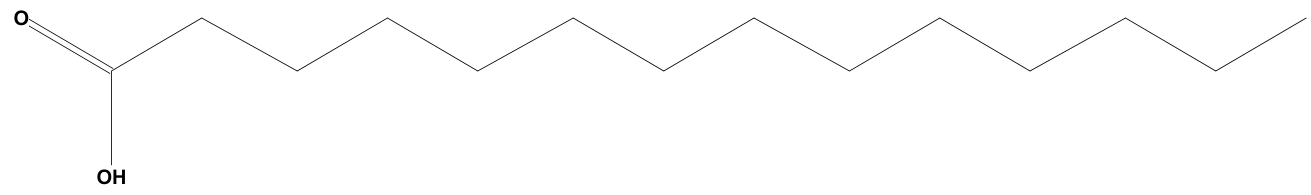 |
| 3-Methyl-2-(2-Oxopropyl) Furan | 138 | C₈H₁₀O₂ | 28.57 | 0.26 | 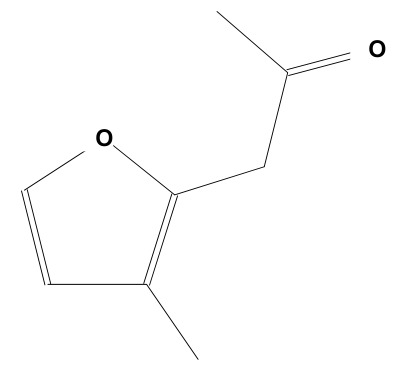 |
| Tetradecanoic Acid, 10,13-Dimethyl-, Methyl Ester | 270 | C₁₇H₃₄O₂ | 30.34 | 0.53 | 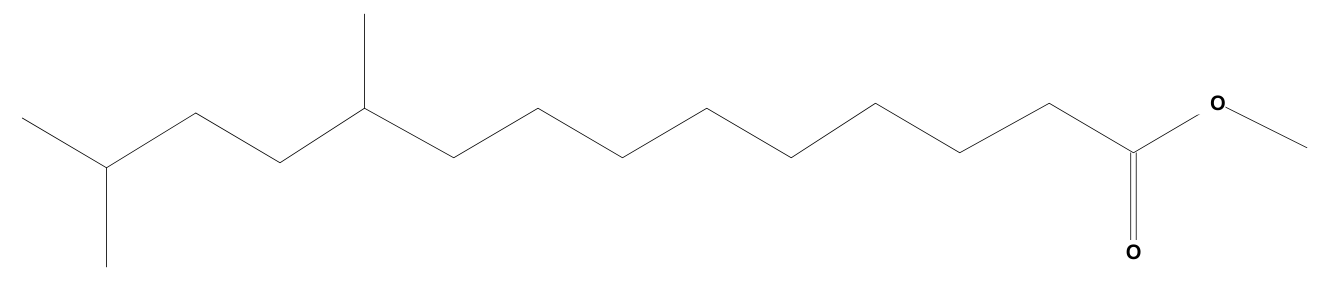 |
| N-Hexadecanoic Acid | 256 | C₁₆H₃₂O₂ | 31.42 | 16.24 | 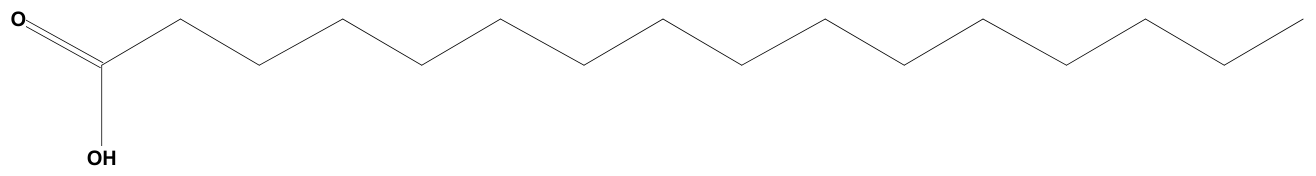 |
| Isopropyl Linoleate | 322 | C₂₁H₃₈O₂ | 33.85 | 0.43 | 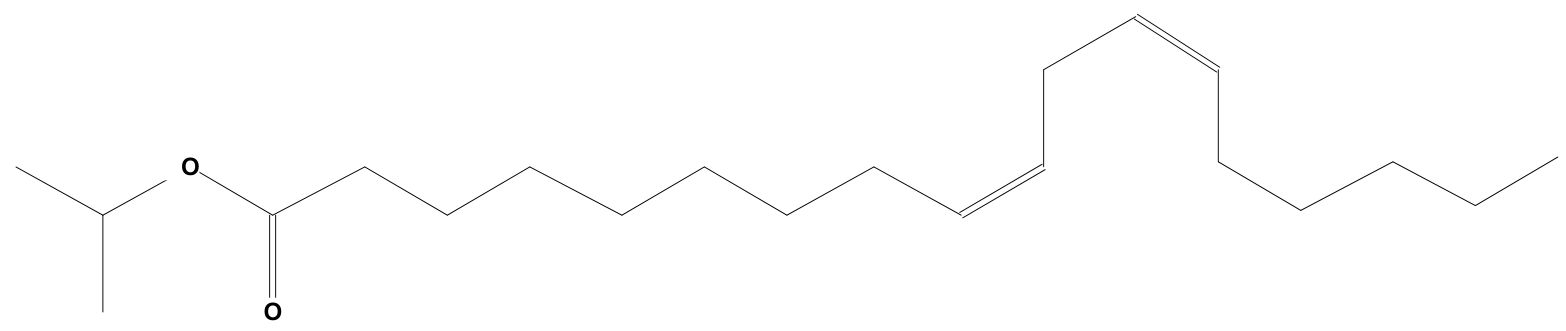 |
| 6-Octadecenoic Acid | 282 | C₁₈H₃₄O₂ | 33.97 | 1.08 | 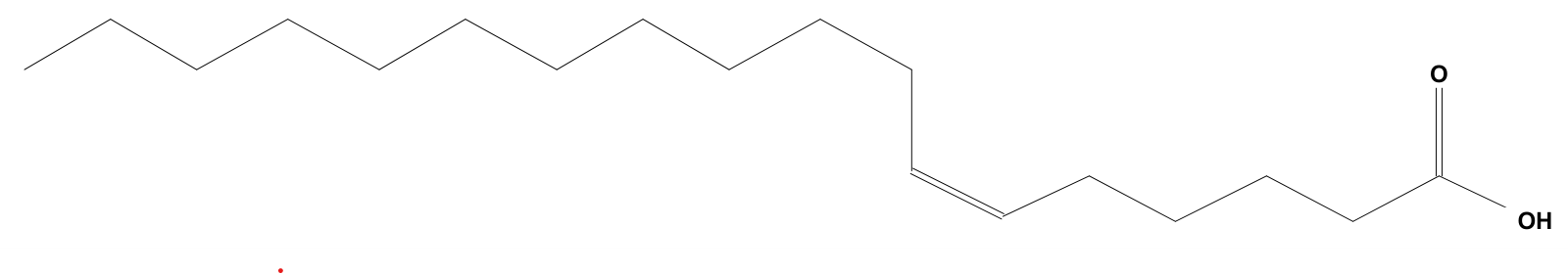 |
| 9-Octadecenoic Acid, (E)- | 282 | C₁₈H₃₄O₂ | 35 | 19.35 | 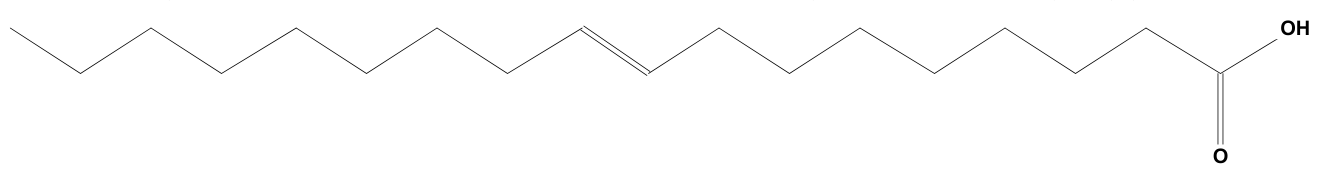 |
| Octadecanoic Acid | 284 | C₁₈H₃₆O₂ | 35.35 | 4.76 | 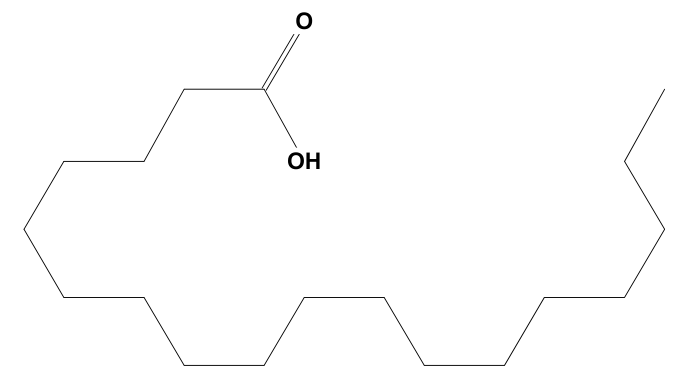 |
| 5-Methyl-Z-5-Docosene | 322 | C₂₃H₄₆ | 36.9 | 0.5 | 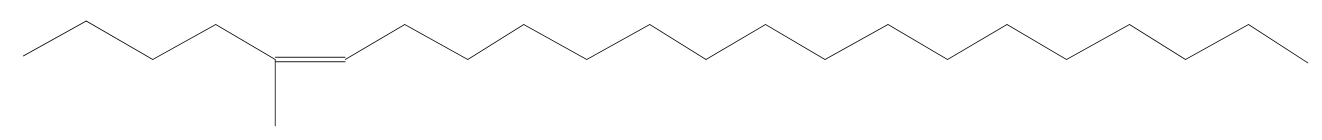 |
| Glycidyl Palmitate | 312 | C₁₉H₃₆O₃ | 37.74 | 3.65 | 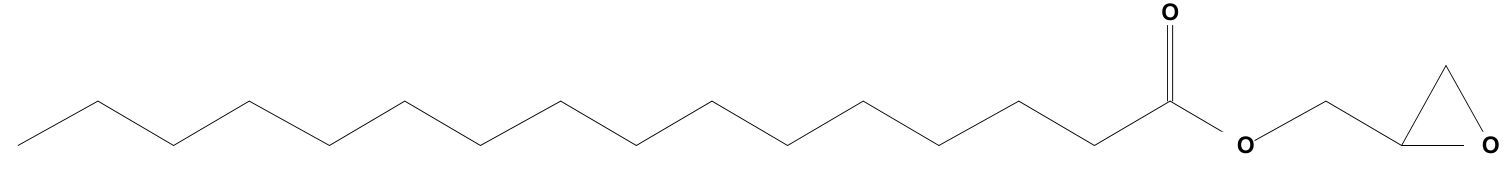 |
| I-Propyl 11,12-Methylene-Octadecanoate | 338 | C₂₂H₄₂O₂ | 38.78 | 1.2 | 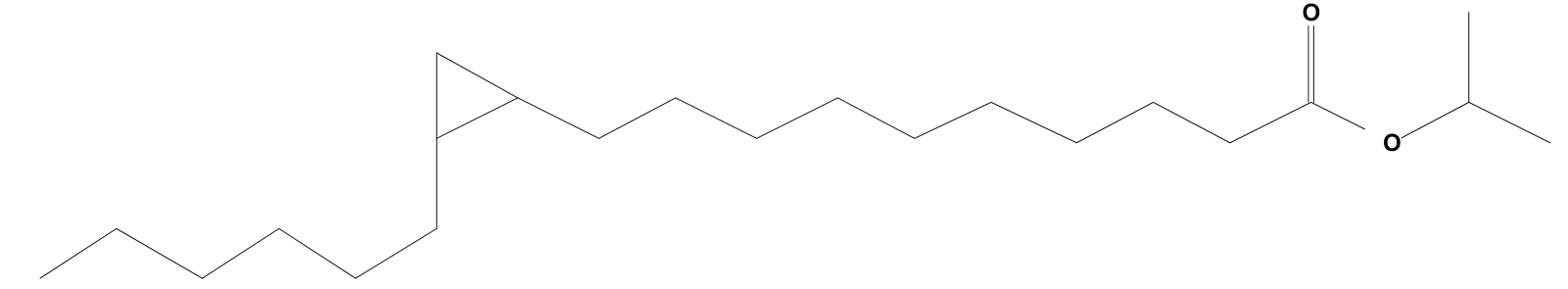 |
| Hexanedioic Acid, Bis(2-Ethylhexyl) Ester | 370 | C₂₂H₄₂O₄ | 39.59 | 1.89 | 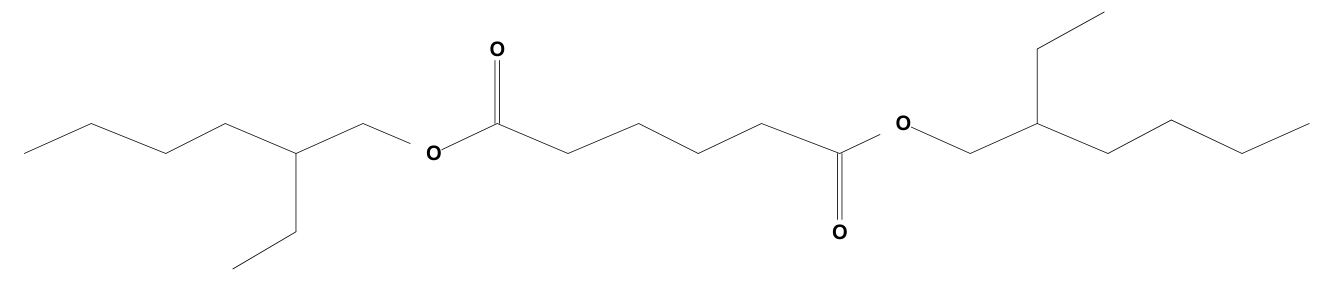 |
| 6-Octadecenoic Acid | 282 | C₁₈H₃₄O₂ | 40.1 | 0.8 | 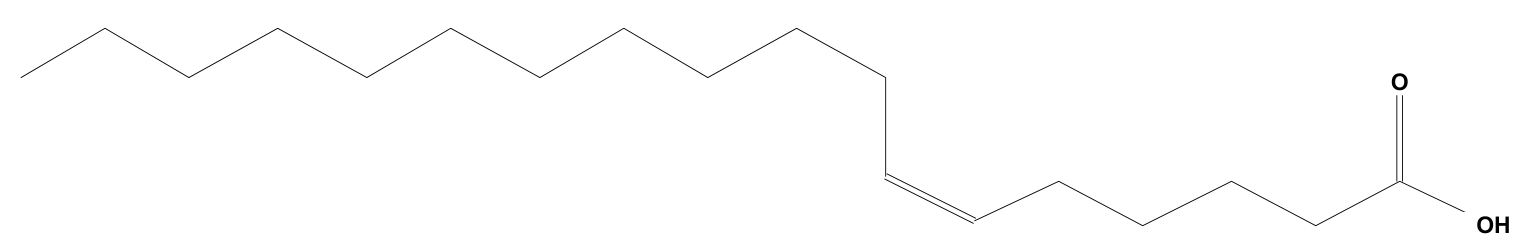 |
| Glycidyl Oleate | 338 | C₂₁H₃₈O₃ | 40.94 | 8.95 | 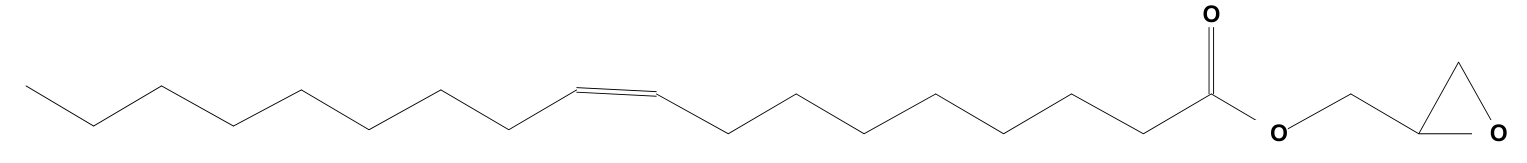 |
| Carbonic Acid, But-3-En-1-Yl Eicosyl Ester | 396 | C₂₅H₄₈O₃ | 41.3 | 3.55 | 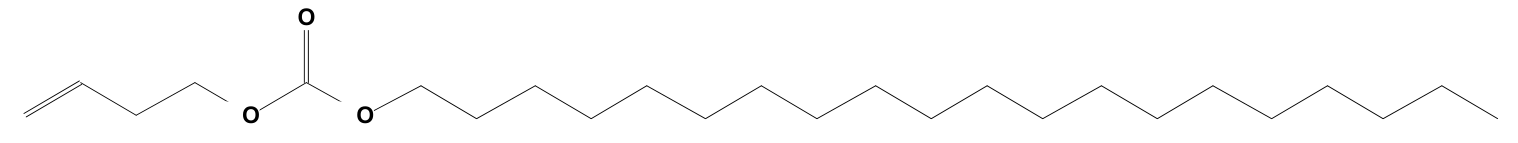 |
| Tetracontane-1,40-Diol | 594 | C₄₀H₈₂O₂ | 41.88 | 0.95 | 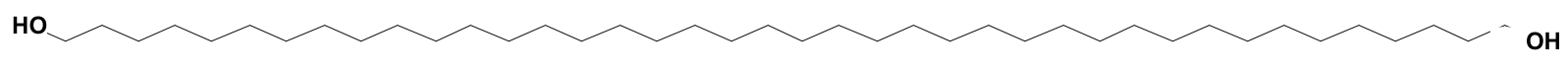 |
| Silane, Dimethyl (Dimethyl (But-3-Enyloxy) Silyloxy) Tridecyloxy- | 402 | C₂₁H₄₆O₃Si₂ | 43.69 | 0.67 | 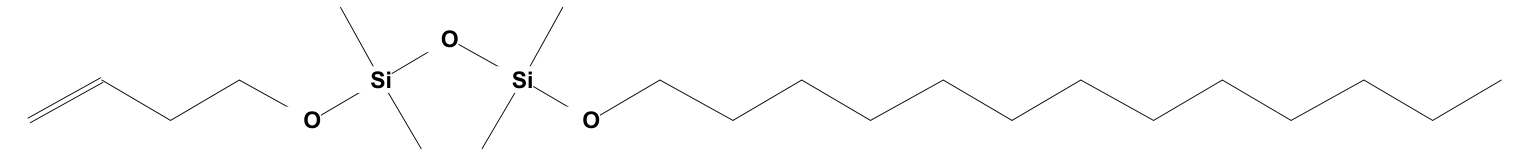 |
| 7-Oxononanoic Acid, Tms Derivative | 244 | C₁₂H₂₄O₃Si | 44.58 | 1.87 | 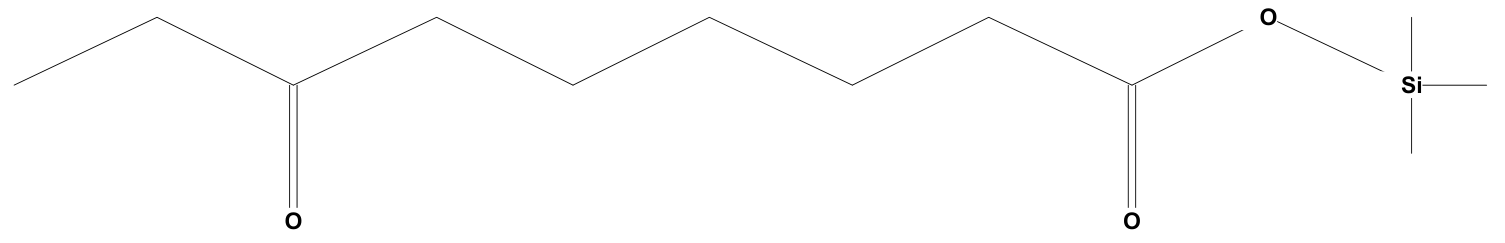 |
| 2,6,10,14-Tetramethyl-7-(3-Methylpent-4-Enylidene) Pentadecane | 348 | C₂₅H₄₈ | 45.03 | 1.43 | 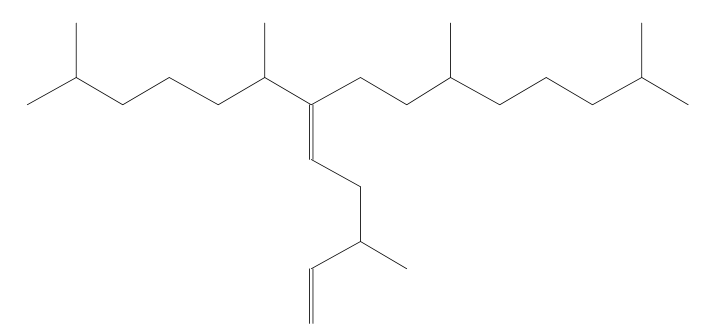 |
| Z, Z-6,27-Hexatriactontadien-2-One | 516 | C₃₆H₆₈O | 45.53 | 3.84 | 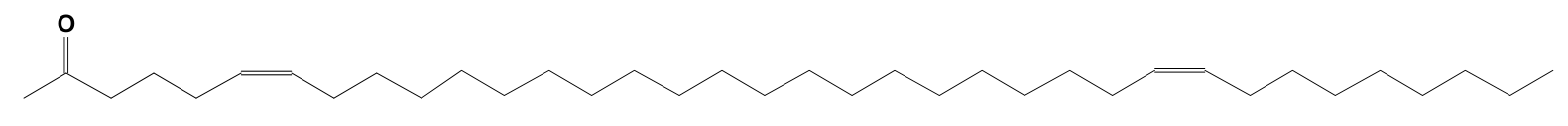 |
| 2,6,10,14-Tetramethyl-7-(3-Methylpent-4-Enylidene) Pentadecane | 348 | C₂₅H₄₈ | 48 | 0.35 | 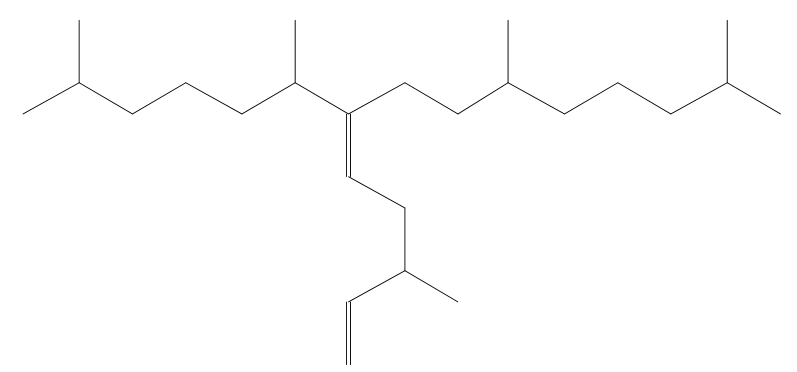 |
| Silane, Dimethyl (Dimethyl (But-3-Enyloxy) Silyloxy) Butoxy- | 276 | C₁₂H₂₈O₃Si₂ | 48.5 | 1.19 | 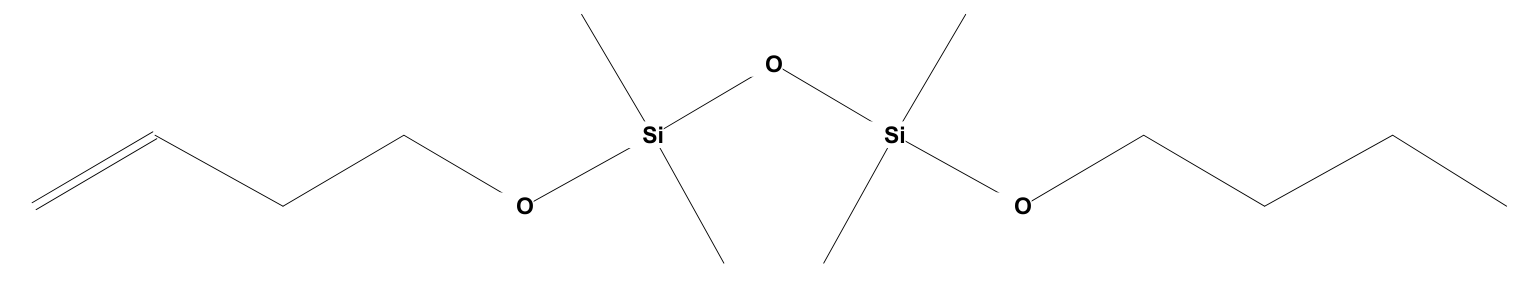 |
| 1,6,10,14,18,22-Tetracosahexaen-3-Ol, 2,6,10,15,19,23-Hexamethyl-, (All-E)- | 426 | C₃₀H₅₀O | 49.79 | 0.96 | 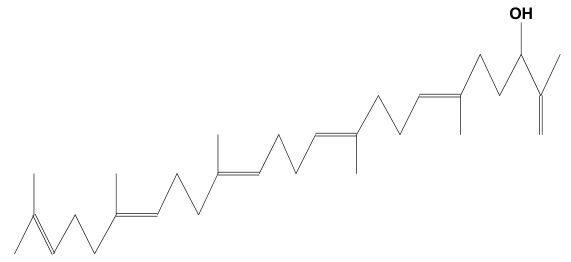 |
| (R)-2,7,8-Trimethyl-2-((3e,7e)-4,8,12-Trimethyltrideca-3,7,11-Trien-1-Yl) Chr | 410 | C₂₈H₄₂O₂ | 51.98 | 1.4 | 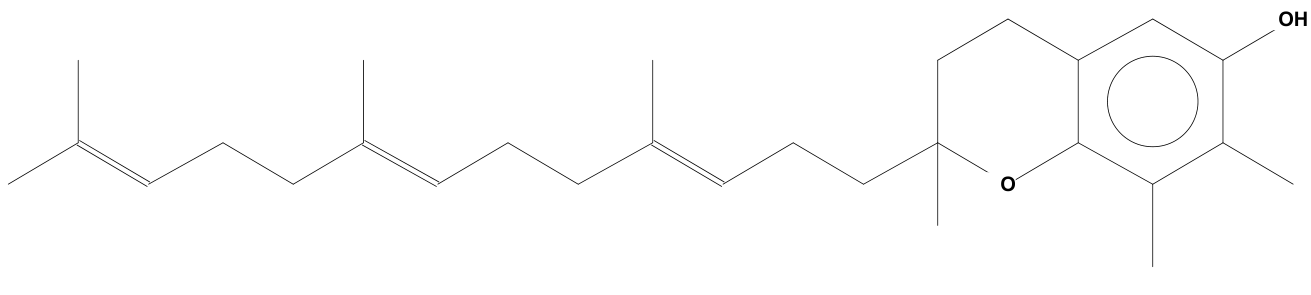 |
| Hentriacontane | 436 | C₃₁H₆₄ | 52.67 | 0.51 | 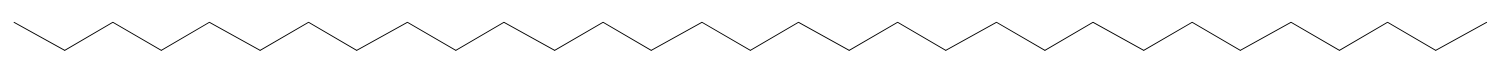 |

| **Table S3:** List of primers and respective sequences | |
| --- | --- |
| **`Primer** | **Sequence** |
| Glucose-6-phosphatase catalytic subunit (G6PC)-F | 5' TGACCTCAGGAACGCCTTCTATG 3' |
| Glucose-6-phosphatase catalytic subunit (G6PC)-R | 5' AGTGTCCAGGACCCACCAATACG 3' |
| Fatty acid synthase (FASN)-F | 5' GGCTCACACACCTACGTATTGG 3' |
| Fatty acid synthase (FASN)-R | 5' TGCTTAATGAAGAAGCATATGGCTT 3' |
| 3-hydroxy-3-methylglutaryl-CoA reductase (HMGCR)-F | 5' AATTGTGTGTGGCACTGTGATG 3' |
| 3-hydroxy-3-methylglutaryl-CoA reductase (HMGCR)-R | 5' GATCTGTTGTGAACCATGTGACTTCT 3' |
